# *Trichophyton rubrum* LysM proteins bind to fungal cell wall chitin and to the N-linked oligosaccharides present on human skin glycoproteins

**DOI:** 10.1371/journal.pone.0215034

**Published:** 2019-04-04

**Authors:** Bibekananda Kar, Pavan Patel, Stephen J. Free

**Affiliations:** Department of Biological Sciences, SUNY University at Buffalo, Buffalo, New York, United States of America; University of California Riverside, UNITED STATES

## Abstract

The *Trichophyton rubrum* genome contains six proteins containing two or more lysin M (LysM) domains. We have characterized two of these proteins, LysM1 and LysM2, and demonstrated that these proteins have the capacity to bind two substrates, chitin and N-linked oligosaccharides associated with human skin glycoproteins. We have characterized the individual LysM domains in LysM1, and shown that the protein contains two functional LysM domains. Each of these domains can bind to chitin, to N-linked oligosaccharides in human skin glycoproteins, and to N-linked oligosaccharides on fungal glycoproteins. We hypothesize that LysM proteins could provide the pathogen with three important functions. First, the *T*. *rubrum* LysM proteins could shield host cell wall chitin from the human immune system. Second, the LysM proteins could shield the pathogen’s glycoproteins from host degradation and immune surveillance. Third, the LysM proteins could help facilitate pathogen adhesion to human skin.

## Introduction

Human-pathogenic dermatophytes exclusively infect keratinized tissues of human skin, nails, and hair [1]. Unlike fungal infections caused by opportunistic fungi in compromised hosts, the dermatophytes are obligate animal pathogens and infect healthy hosts. Dermatophytes seldom colonize beyond the epidermis. However, in patients with a CARD9 deficiency dermatophytes, have been reported to cause subcutaneous infections and to spread to the lymph nodes [2]. Superficial dermatophyte infections are very common, and 10–20% of the worldwide human population suffer from dermatophyte infections [1]. These infections are often very difficult to treat and have a high frequency of reoccurrence [3]. *Trichophyton rubrum* is the most common fungal dermatophyte and is known to account for 60% of all clinical dermatophyte infections. The fungus causes a variety of chronic skin infections including toenail infections (onychomycosis) athlete’s foot (tinea pedis), ringworm (tinea capitis), and jock itch (tinea cruris).

The significance of the cell wall for fungal survival and pathogenicity has been well-established. The cell wall maintains the shape and integrity of the fungal cell, provides mechanical strength to the cell, and protects the cell from external environmental stresses [4–6]. In pathogenic fungi, the cell wall also mediates pathogen adhesion to tissues to initiate colonization and infection. The fungal cell wall is also the source of pathogen-associated molecular patterns (PAMPs) including chitin, glucans, and mannans [7–11]. These are recognized by the innate immune system, which provides protection from fungal pathogens. Pathogenic fungi have evolved a variety of cell wall-based mechanisms to help them evade host immune systems [8, 9, 11, 12].

LysM motifs are carbohydrate-binding modules found in bacteriophages, bacteria, fungi, plants and animals. LysM domains are comprised of a characteristic βααβ structure with a shallow groove for carbohydrate binding on one surface. The motif was first observed and characterized in bacteriophage lysin proteins and a bacterial muramidase (peptidoglycanase) [13]. The domain has been subsequently found in proteins from many organisms. LysM domains have been shown to bind to *N*-acetylglucosamine-containing carbohydrates, such as chitin and bacterial peptidoglycan. LysM domains are found in enzymes for the degradation or modification of peptidoglycans and chitin, where they are thought to function in targeting the proteins to its substrate. The structure of the motif and the molecular basis for peptidoglycan and chitin recognition by LysM domains have been studied [13–18]. LysM domain proteins play particularly important roles in plant/pathogen interactions and in plant/symbiont interactions [19–21]. Plants have LysM receptor kinase proteins that allow the plant to recognize the presence of bacterial and fungal pathogens via the receptor’s LysM domain. The LysM receptor kinases activate plant immune responses through their intracellular kinase domains [21]. Symbiotic relationships between plants and the arbuscular mycorrhiza and between legumes and rhizobial bacteria are mediated through related LysM domain receptor kinases that signal to mediate the symbiotic relationship [19, 20]. The plant LysM receptor kinases involved in these symbiotic relationships recognize lipochitin oligosaccharides released by the bacteria and mycorrhiza.

Plant pathogenic fungi have been shown to produce LysM proteins that bind to the fungal cell wall chitin and to chitin fragments released from the cell wall by plant chitinases [22–27]. The released chitin activates the plant innate immune response. By binding to the chitin fungal LysM proteins can protect the cell wall chitin from chitinase digestion. The LysM proteins can also bind the released chitin fragments and shield them from the plant immune system. Thus, these fungal LysM proteins help the fungus evade detection by the host plant and are important effectors of plant disease. Interestingly, these fungal LysM proteins often contain multiple LysM domains.

In the *T*. *rubrum* genome, the LysM gene family of cell wall proteins has undergone a dramatic expansion not seen in other closely related fungi [28]. This suggests that these proteins may play an important role in the *T*. *rubrum* infection process. Here we characterize the *T*. *rubrum* LysM1 and LysM2 proteins (encoded by genes TERG_05627 and TERG_01873 respectively). These two proteins were chosen for the analysis as representatives of the B clade of LysM proteins, which are proteins lacking a chitinase domain [28]. The two proteins are not closely related paralogs, and were chosen to represent the diversity that might be present in the LysM B clade. Both LysM1 and LysM2 have three putative LysM domains. An analysis of the amino acid sequence suggests that one of the putative LysM domains in LysM1 is a truncated non-functional domain while the other two are functional domains. Sequence analysis of the three LysM domains in LysM2 indicates that all three domains are likely to be functional domains. To characterize these proteins, we cloned and expressed them in *Neurospora crassa* (a model fungus) and in *E*. *coli*. We demonstrate that LysM1 and LysM2 bind to chitin and that the proteins bind to the *N*. *crassa* and *T*. *rubrum* cell walls. We show that the two functional LysM domains from LysM1 bind to chitin. We also show that these same two domains bind to human skin lysate glycoproteins. LysM1 and LysM2 were not able to bind to human skin lysate glycoproteins that had been treated with PNGase F, demonstrating that LysM1 and LysM2 recognize the N-linked oligosaccharides present on the skin glycoproteins. We hypothesize that LysM1 and LysM2 may recognize the chitobiose structure found at the base of the N-linked oligosaccharide. Our findings suggest that LysM1 and LysM2 could function to mask the pathogen’s cell wall components (chitin and/or glucans) from the human innate immune system. The LysM proteins could also function as adhesion proteins that recognize human skin glycoproteins and facilitate adhesion to them.

## Materials and methods

### Strains and growth conditions

*T*. *rubrum* strain MYA-4607 was obtained from the American Type Culture Collection and maintained on Sabouraud Medium (40g/l glucose, 01 g/l peptone, pH 5.6). *N*. *crassa* strains were from the Fungal Genetics Stock Center (Manhattan, KS) and were maintained on Vogel’s minimal medium [29]. *N*. *crassa* mating was carried out on Whatmann 3MM paper in synthetic mating medium and single ascospores were isolated on sorbose agar plates [29].

### Chemicals and antibodies

Isopropyl β-D-1-thiogalactopyranoside (IPTG), phenylmethylsulphonyl fluoride, and maltose were purchased from Sigma-Aldrich Corp. (St. Louis, MO). Amylose resins, chitin magnetic beads, restriction enzymes, Phusion polymerase, PNGase F, and T4 DNA ligase were purchased from New England Biolabs (Ipswich, MA). Antibody directed against Maltose Binding Protein (product E8032) was obtained from New England Biolabs and used according to the manufacturer’s instructions. The AlexaFluor 488 and AlexaFluor 633-tagged goat anti-mouse antibody (products A11029 and A21126) was purchased from ThermoFisher Scientific (Waltham, MA). Monoclonal mouse antibody directed against GFP (product # 11814460001) was purchased from Sigma-Aldrich (St. Louis, MO). Western blots were carried out with the ECL kit from BioRad (Hercules, CA). Human whole skin lysate was purchased from Zyagen (San Diego, CA).

### Sequence analysis

The amino acid sequence of the *T*. *rubrum* LysM1 (TERG_05627, accession # EGD89384.2) and LysM2 (TERG_01873, accession #EGD95603.1) proteins were retrieved from NCBI and used in the BLASTp program for homology searches. For analysis of site conservation, multiple sequence alignment was carried out using Clustal Omega webserver taking default parameters (https://www.ebi.ac.uk/Tools/msa/clustalo/). Secondary structure of LysM protein was predicted by using SOPMA (https://npsa-prabi.ibcp.fr/cgi-bin/npsa_automat.pl?page=/NPSA/npsa_sopma.html) and Jpred (http://www.compbio.dundee.ac.uk/jpred/) webserver.

### Gene synthesis, cloning, expression, and purification of recombinant *T*. *rubrum* LysM proteins

The codon-optimized versions of *T*. *rubrum* LysM1 and LysM2 in which the codon usage was optimized for expression in *N*. *crassa*, was chemically synthesized by Genewiz Inc. (South Plainfield, NJ) and cloned into the *E*. *coli* vector pUC57-amp resulting in the plasmid vectors pUC57-LysM1 and pUC57-LysM2. For the expression in *N*. *crassa*, the pUC57-LysM1 and pUC57-LysM2 vectors were digested with the XbaI and BamHI restriction enzymes, and the fragments containing the LysM coding sequences were ligated into corresponding restriction sites of pMF272 (Bowman et al., 2009) to generate the pKB01 and pKB02 plasmids (S1 and S2 Figs). The pKB01 and pKB02 plasmids contain a region from the *N*. *crassa his-3* gene, the *N*. *crassa ccg-1/grg-1* promoter, the LysM1 and LysM2 coding regions respectively, the green fluorescent protein (GFP) coding region, and a region from the *his-3* 3’UTR. During transformation, the plasmid sequences from the *his-3* gene and from its 3’ flanking region allow for the insertion of the plasmid into the *N*. *crassa* genome via a homologous recombination event. When transforming the a *his-3* mutant isolate, the insertion of the plasmid generates a wild type *his-3* allele and allows for the selection of transformants as histidine prototrophs. The pKB01 and pKB02 plasmids were transformed into *N*. *crassa* conidia and transformants were isolated on Vogel’s sorbose agar medium [30]. The sequencing of PCR products amplified from the transformants demonstrated that the transformants contained the chimeric LysM1::GFP and LysM2::GFP sequences.

In an effort to get secreted LysM1::GFP and LysM2::GFP to facilitate further characterization of the proteins, the transformants were mated with the *Δoch-1* mutant [29]. The *Δoch-1* mutant has been shown to be unable to attach cell wall glycoproteins into the cell wall matrix and releases these proteins into the medium [31]. Single ascospores progeny from the mating were isolated and *Δoch-1*, *LysM1*::*GFP* and *Δoch-1*, *LysM2*::*GFP* isolates were obtained. For the expression of LysM1 and LysM2 in *E*. *coli*, the gene sequence encoding the genes were amplified by PCR from the pUC57-LysM1 and pUC57-LysM2 vectors using gene-specific primers (S1 Table) and cloned in pMAL-p5X vector to generate the pMAL-p5X-LysM1 and pMAL-p5X-LysM2 plasmids which encode the chimeric maltose binding proteins MBP:: LysM1 and MBP::LysM2. The recombinant plasmids were transformed into high efficiency NEB Express competent *E*. *coli* cells for high level expression of the protein (New England Bio Labs, Ipswich, MA). The expression of the MBP::LysM1 and MBP::LysM2 proteins was induced by the addition of 300μM IPTG and the cultures were kept at 18°C for sixteen hours while the chimeric proteins were produced. The chimeric proteins were purified by using Amylose bead affinity chromatography according to the manufacturer’s instructions (New England Biolabs, Ipswich, MA). The purity of the recombinant MBP::LysM1 and MBP::LysM2 proteins were analyzed by electrophoresis in a 12% SDS-PAGE. The purified fractions were pooled, dialyzed, and concentrated by using an Amicon Ultra concentrator with a cut off value of 10 kDa (Millipore, Bedford, MA, USA).

### Construction and expression of vectors containing individual LysM1 domains

To characterize the individual LysM1 domains present in LysM1, plasmids encoding chimeric MBP::single domain or MBP::double domain proteins were prepared. Various portions of *T*. *rubrum* LysM1 gene were amplified by PCR from pUC57-LysM1, and cloned into pMAL-p5X in order to generate plasmids encoding chimeric MBP::LysM1 domain(s) proteins. Five plasmid constructs were prepared. Three of these encoded MBP attached to each of the individual domains. The last two constructs encoded MBP attached to domains 1 and 2 and MBP attached to domains 2 and 3. These individual domain plasmids were generated by using the primers shown in S1 Table to PCR amplify the regions encoding the various domain(s), digesting the PCR products with NdeI and BamHI, and inserting the PCR products into pMAL-p5X. The chimeric MBP::individual domain proteins were expressed and purified as described above for the full-length LysM1. The chitin binding activity and keratinocyte lysate binding activity described below were used to evaluate and characterize the individual LysM domains.

### Confocal microscopy

Fluorescence confocal microscopy was used to assess the location of GFP-tagged LsyM1and GFP-tagged LysM2 in *N*. *crassa*. The location of MBP-tagged LysM1 added to *T*. *rubrum* and *N*. *crassa* hyphae was also assessed by fluorescence confocal microscopy. For looking at LysM1::GFP and LysM2::GFP that was expressed in *N*. *crassa*, the cells were grown on agar slants and the conidia (asexual spores) were harvested in water and examined under the microscope. For localization studies using the MBP::LysM1, *T*. *rubrum* and *N*. *crassa* hyphae were grown in liquid Sabouraud and Vogel’s media, harvested by centrifugation (6,000 x g for 10 minutes), washed in PBS buffer, and incubated with purified MBP::LysM1, MBP::LysM2, or MBP proteins. Following the incubation step, the cells were washed three times in PBS, and incubated with mouse monoclonal antibody directed against MBP (New England Biolabs). After the incubation with the anti-MBP antibody, the cells were washed three times with PBS and incubated with goat anti-mouse IgG conjugated with Alexa Fluor 488 or Alexa Fluor 633 (ThermoFisher Scientific). The cells were then washed with PBS and examined under a Zeiss LSM710 Confocal Laser Scanning microscope. All the incubation steps were at 25°C for 1 hour.

### Chitin binding assay

Chitin magnetic beads (New England Biolabs, Ipswich, MA) were used to assess the ability of LysM1, LysM2, and the individual LysM domains from LysM1 to bind to chitin. The chitin beads were first washed three times with column binding buffer (20 mM Tris-HCI, pH 8.0, 500 mM NaCl, 5% glycerol, 1 mM EDTA, 0.5% Tween 20). 20 μl of chitin magnetic beads (50% slurry) were added per ml of *E*. *coli* cell lysates containing MBP, MBP::LysM1, MBP::LysM2, or MBP::LysM domain(s) proteins. Magnetic beads and the cell lysate samples were incubated at 25°C on a rotator for 1 hour. The magnetic beads were then collected, washed three times with column binding buffer, and the bound proteins were eluted from the beads by boiling the samples in SDS PAGE sample loading buffer. The samples were subsequently analysed by SDS-PAGE and Western blotting.

### LysM1 binding experiments with skin lysate, *N*. *crassa* cell wall proteins, and *N*. *crassa* cellular lysate

The binding of MBP, MBP::LysM1, MBP::LysM1 domains, and MBP::LysM2 to proteins found in human skin lysate was assessed by Western blot experiments. Aliquots of human skin lysate (Zyagen, San Diego, CA) containing 10 μgr of protein cell lysate were subjected to 12% SDS-PAGE under denaturing conditions. After SDS-PAGE, proteins were electro-transferred to nitrocellulose membrane (Invitrogen, Carlsbad, CA) in the presence of 20 mM Tris-HCl, pH 8.3, 192 mM glycine, 0.1% SDS, and 20% methanol. The membranes were then blocked with blocking buffer (PBS containing 0.1% Tween 20 and 5% non-fat milk) overnight at 4°C. Thereafter, the membrane were incubated with 50 μgr purified MBP::chimeric proteins in blocking buffer for one hour at 25°C. Subsequently, the membranes were washed three times for 10 min with blocking buffer and incubated with monoclonal antibody directed against MBP (NEB, Ipswich, MA) in blocking buffer for 1 hour at 25°C. The membranes were then washed three times with blocking buffer for 10 min and incubated for 1 hour at 25°C with secondary antibody coupled to horse radish peroxidase. The presence of the antibodies was visualized on a BioRad gel doc instrument using the BioRad clarity Western ECL substrate system (BioRad, Hercules, CA).

The binding of LysM1 to fungal glycoproteins was also assessed by Western blot type experiments. Cell wall proteins were obtained from the growth medium in which the *N*. *crassa Δgel-1*, *Δgel-2*, *Δgel-5* mutant was grown. This mutant releases cell wall proteins to the growth medium [32]. The growth medium was collected by passing the culture over a Buchner funnel and the cell wall glycoproteins were precipitated with an ammonium sulphate precipitation [32]. Total *N*. *crassa* cellular protein was obtained by growing the wild type strain in Vogel’s 2% sucrose liquid medium for 24 hours at 30°C and collecting the cells on a Buchner funnel [29]. A cellular extract was prepared by grinding the cells in a mortar and pestle under liquid nitrogen and extracting the proteins into PBS containing 1% SDS. The secreted cell wall proteins and total cell extract proteins were subjected to SDS PAGE and the ability of LysM1 to bind to the glycoproteins was assessed as described above for skin lysate proteins.

## Results

### Analysis of the LysM1 and LysM2 coding sequences

**Fig 1:**
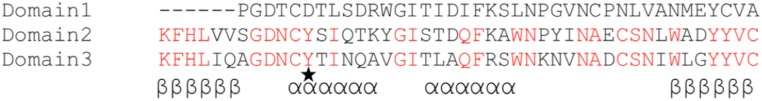
Sequence analysis of the LysM domains in LysM1. The Clustal Omega sequence alignment tool was used to align the LysM domain sequences in the LysM proteins. Note that the first LysM domain in LysM1 is missing the first six amino acids, which are needed for the first β in the βααβ structure found in LysM proteins. Amino acids in red are those that are identical in domain 2 and domain 3. The β and α denote areas involved in forming β sheets and α helices.

When the amino acid sequence for LysM1 (TERG_05627, accession # EGD89384.2) was analysed, we noted that a methionine found at location 36 was the most likely start of translation because a signal peptide sequence lies immediately downstream of this methionine. The 262 amino acid sequence beginning with the methionine was used for the synthesis of a codon-optimized version of LysM1 as described in the Materials and Methods. Analysis of the LysM1 amino acid sequence showed the presence of three regions of LysM sequence homology (Fig 1). The first of these domains was a truncated domain lacking the first 6 amino acids, while the second and third domains were full-length domains. LysM domains have a characteristic βααβ structure, and the first domain lacks the first β strand (Fig 1). A previous analysis of a *Pteris ryukyuensis* chitinase LysM domain showed the involvement of the tyrosine residue in the first α helix in carbohydrate binding [16]. In the first LysM1domain, this tyrosine is lacking. Based on the sequence analysis of LysM1, it is likely that the first LysM1 domain is non-functional while the second and third domains are likely to be functional domains.

The LysM2 protein contains three LysM domains and analysis of these domains suggested that all three are functional. The optimized version of LysM2 was made using the amino acid sequence given for LysM2 (TERG_01873 accession #EGD95603.1).

### Cloning and expression of LsyM1::GFP and LysM2::GFP in *N*. *crassa*

**Fig 2:**
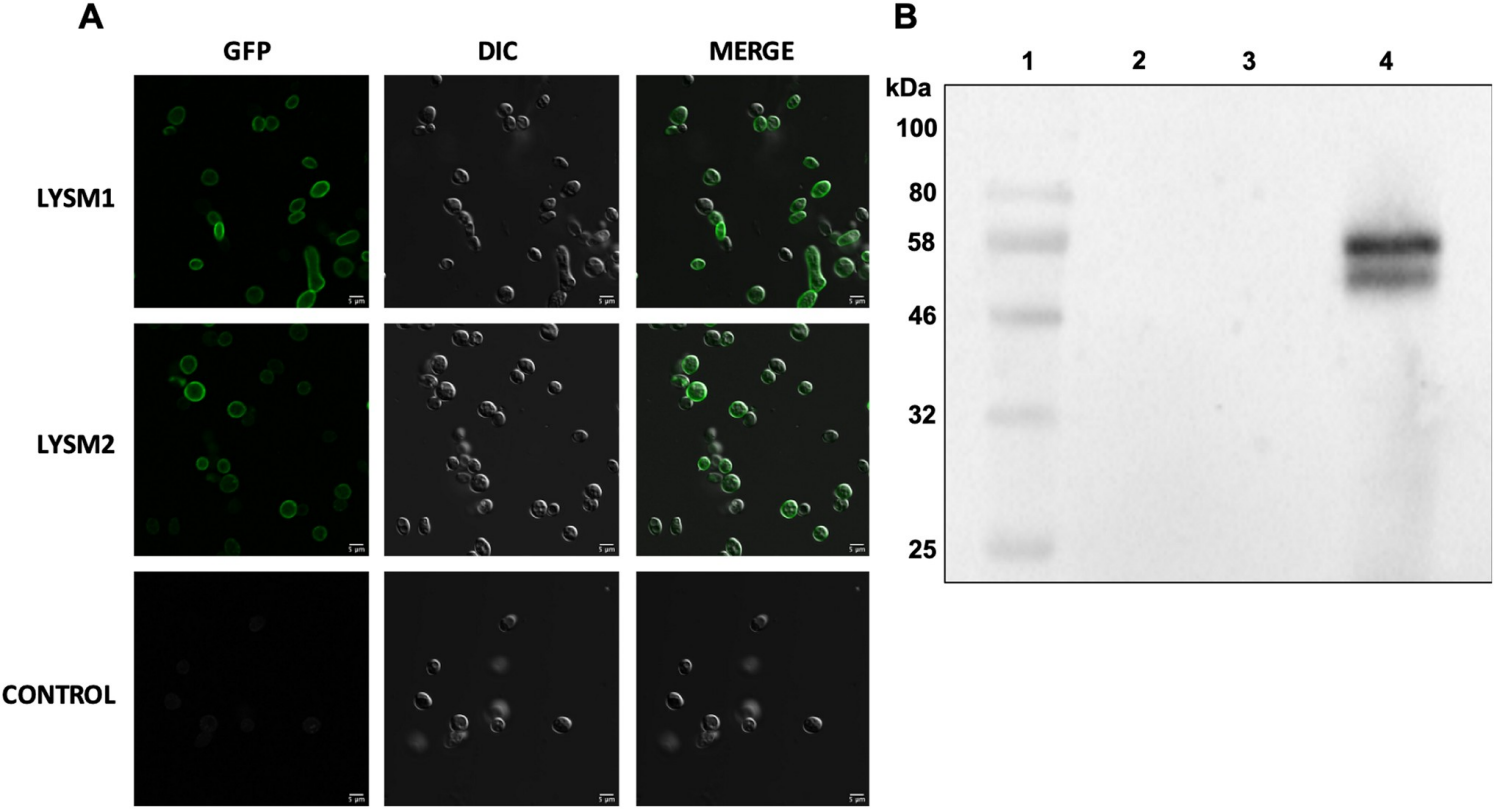
LysM1 and LysM2 bind in a non-covalent manner to *N*. *crassa* cell walls. A) *N*. *crassa* conidia from a transformant expressing LysM1::GFP (top row), from a transformant expressing LysM2::GFP (middle row), and from the non-transformed parental strain (bottom row) were examined by confocal microscopy. The left panels show the GFP images, the middle panels show the DIC images, and the right panels show the merged images. B) A Western blot analysis of LysM1::GFP released from a *Δoch-1* isolate. Lane 1 contains molecular weight markers. Lane 2 contains a sample of the growth medium. Lane 3 contains proteins released from an isolated cell wall fraction with a PBS wash. Lane 4 contains proteins released from the isolated cell wall fraction by a wash with PBS containing 1% SDS.

GFP-tagged codon-optimized versions of LysM1 (encoded by gene TERG_05627) and LysM2 (encoded by gene TERG_01873) were used to transform *N*. *crassa* as described in Materials and Methods. Examination of the fungal transformants showed that the protein was localized to the cell wall. Fig 2 shows LysM1::GFP expressing conidia, a cell type in which the promoter used to drive LysM1::GFP expression is highly expressed, with GFP-tagged LysM1 in the cell wall. Transformants expressing LysM2::GFP also showed that the GFP-tagged LsyM2 was located in the cell wall (Fig 2). These results demonstrate that the signal peptides in the proteins target the protein into the secretory pathway and the proteins become incorporated into the cell wall.

With the intent of obtaining LysM1::GFP that was released into the growth medium as a soluble protein, LysM1::GFP and LysM2::GFP expressing transformants were mated with the *Δoch-1* mutant. The *Δoch-1* mutant has been previously shown to be unable to incorporate cell wall proteins into the cell wall and releases cell wall proteins into the medium [31]. *Δoch-1*, LysM1::GFP and *Δoch-1*, LysM2::GFP isolates were obtained from these matings, and we examined the location of tagged proteins in these strains. We found that the LysM1::GFP and LysM2::GFP proteins remained associated with the cell wall (S3 Fig). Fig 2B shows that LysM1 was not present in the growth medium from the *Δoch-1*, LysM1::GFP isolate (lane 2), and is not released from the purified cell walls by a PBS wash (lane 3). However, LysM1::GFP was released from the purified cell walls by a wash containing 1% SDS in PBS (lane 4). These results indicate that the association between the wall and LysM1 was not through a covalent bond. Because the *Δoch-1* mutant cell wall is unable to incorporate glycoproteins into the cell wall matrix, these results strongly suggest that LysM1 and LysM2 are associating with the chitin or glucans present in the *N*. *crassa* cell wall. We further found that we could release LysM1 and LysM2 from the cell walls of our original *N*. *crassa* transformants by treatment with 1% SDS (not shown), which further corroborates the conclusion that LysM1 and LysM2 are not covalently attached to the wall but are bound to the fungal cell wall glucan/chitin matrix.

### Expression of LysM1 and LysM2 in *E*. *coli* and demonstrating that they have chitin binding activity

**Fig 3:**
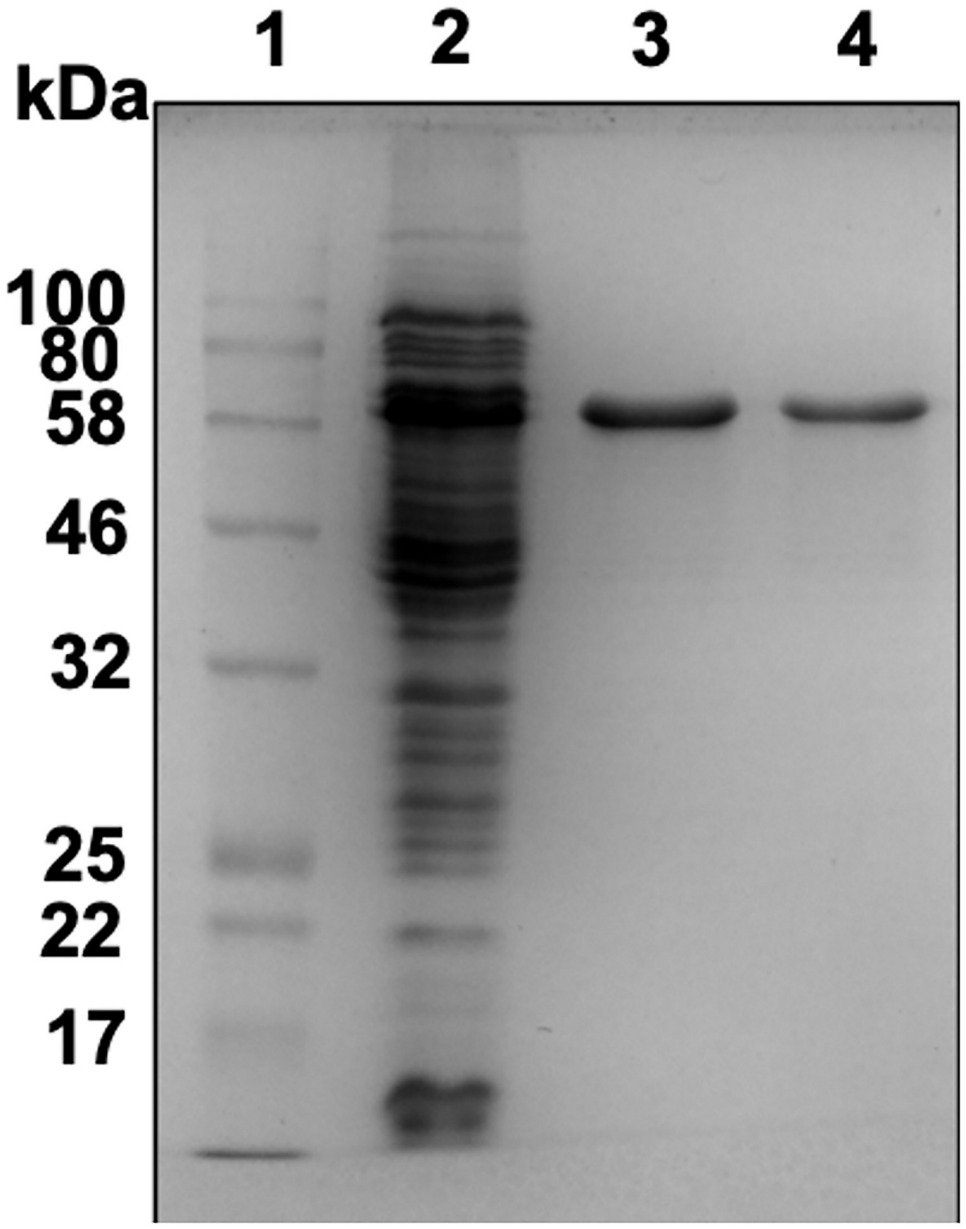
Purification of MBP::LysM1. A Comassie blue stained SDS PAGE gel demonstrating the purification of MBP::LysM1. Lane 1 contains molecular weight markers. Lane 2 contains *E*. *coli* cell lysate. Lanes 3 and 4 show fractions of purified protein from the amylose affinity purification column used to purify the protein.

To obtain purified soluble LysM1 for biochemical characterization, the LysM1 and LysM2 coding sequences were cloned into the pMAL-p5X vector to create chimeric maltose binding protein (MBP) constructs. We created the pBK03 and pBK04 plasmids, which encode MBP::LysM1 and MBP::LysM2 respectively. *E*. *coli* transformants containing pBK03 and pBK04 were used to produce the chimeric proteins. *E*. *coli* lysates containing the chimeric proteins and control lysates with MBP were used in some of our experiments. For other experiments, the proteins were purified on an amylose resin in accordance with the manufacturer’s instructions. Fig 3 shows an SDS PAGE gel demonstrating the purification of MBP::LysM1. Similar results were obtained for the purification of LysM2 (S4 Fig). The chimeric proteins were used to characterize the binding activities of LysM1 and LysM2, with MBP serving as a control.

**Fig 4:**
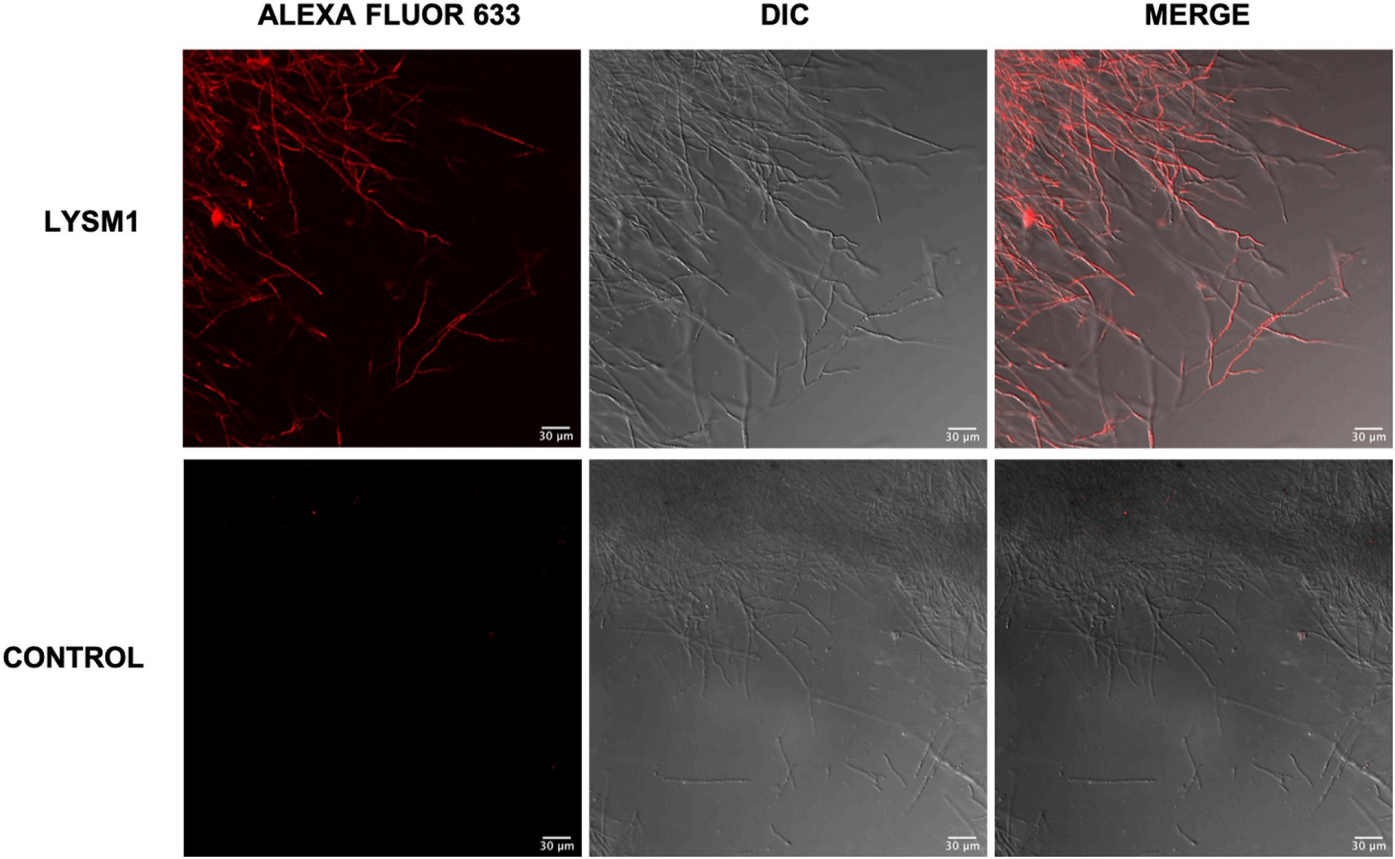
LysM1 binds to *T*. *rubrum* cell walls. *T*. *rubrum* hyphae were incubated with purified MBP::LysM1 and the binding of LysM1 to the cells was visualized with a fluorescent antibody protocol using antibody directed against MBP. The top row shows cells stained for the binding of MBP:: LysM1 and the bottom row contains cells stained for the binding of the MBP control. The left panels show the fluorescent images. The middle panels show the DIC images. The right panels contain the merged images.

We first asked the question of whether LysM1 and LysM2 could bind to the cell walls of *T*. *rubrum* and *N*. *crassa* hyphae. Hyphae cells grown in liquid medium were incubated with purified MBP, MBP::LysM1, and MBP::LysM2 and the ability of LysM1 and LysM2 to bind to the cells was assessed with the anti-MBP antibody staining protocol described in Materials and Methods. We found that *E*. *coli*-produced LysM1 and LysM2 were able to bind to the *N*. *crassa* and *T*. *rubrum* cell walls. Fig 4 shows the chimeric LysM1 binding to the *T*. *rubrum* cells. The control experiment, in which MBP was used instead of the chimeric MBP::LysM1, showed that MBP does not bind to the hyphae. Similar results were obtained with *N*. *crassa* cells (S5 Fig). We conclude that LysM1 and LysM2 specifically bind to *T*. *rubrum* and *N*. *crassa* cell walls. We hypothesize that the binding is likely to be to the cell wall chitin or glucans based on the fact that LysM domains recognize carbohydrates, and on the results shown in Fig 2B demonstrating that LysM1 binds to the cell wall of the *N*. *crassa Δoch-1* mutant, which is largely devoid of cell wall protein.

**Fig 5:**
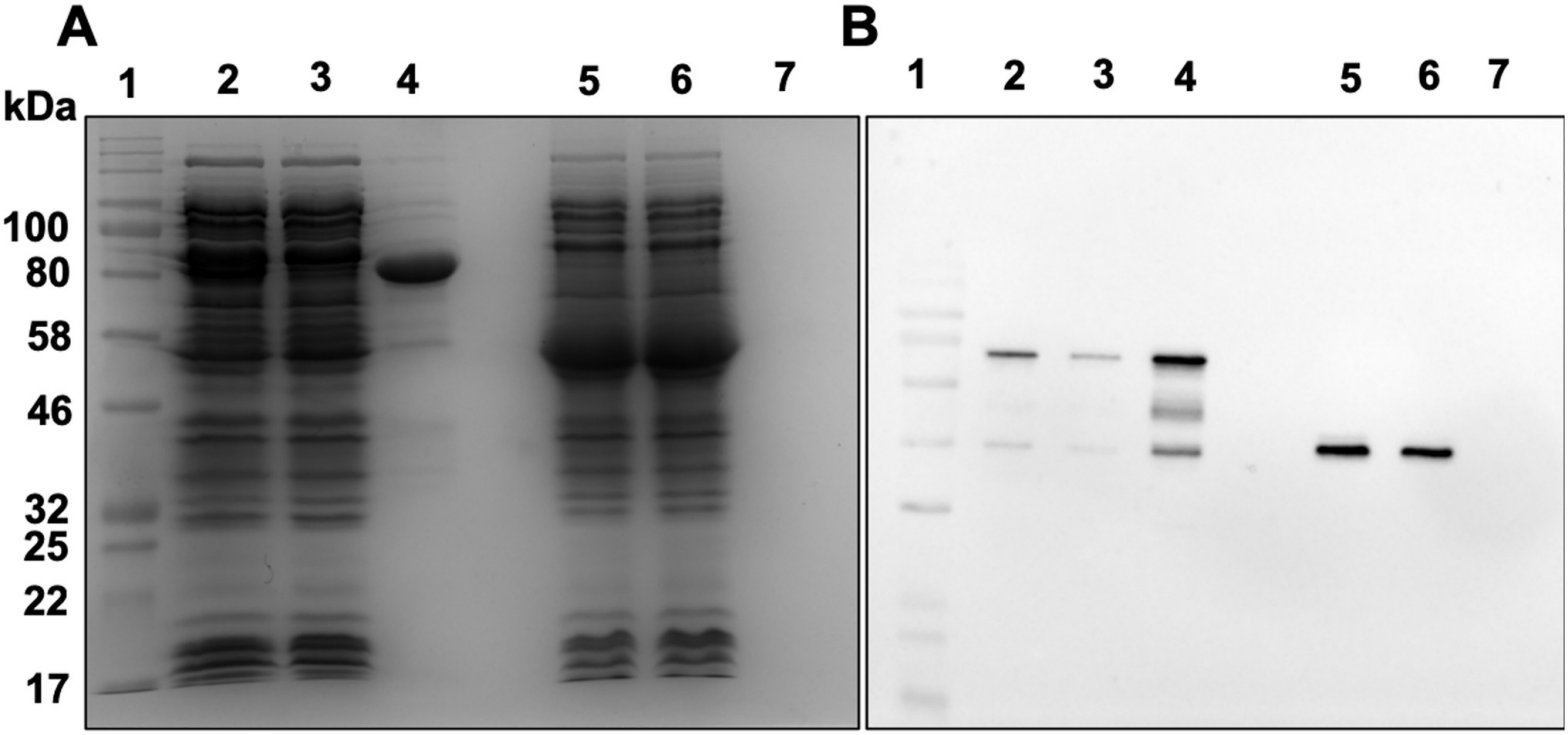
LysM1 binds to chitin. *E*. *coli* lysates containing MBP::LysM1 or MBP were incubated with chitin magnetic beads to purify chitin binding proteins. A Coomassie Blue-stained SDS PAGE gel experiment (panel A) and a Western blot experiment (panel B) were used to assess the ability of MBP::LysM1 (lanes 2–4) and MBP (lanes 5–7) to bind to the beads. In both panels, lane 1 contains molecular weight markers. Lanes 2 and 5 contain a sample of the lysate. Lane 3 and 6 contain lysate after the incubation with the chitin magnetic beads. Lanes 4 and 7 show the proteins bound to the chitin magnetic beads. The MBP::LyM1 protein shows some evidence of proteolysis with smaller molecular weight bands being present. Note that MBP::LysM1 binds chitin (lane 4) while the MBP control does not (lane 7).

To directly assess whether LysM1 and LysM2 bind to chitin, *E*. *coli* lysates containing MBP::LysM1, MBP::LysM2, and MBP were incubated with chitin magnetic beads (Fig 5). Following the incubation step, the beads were collected, washed, and the bound protein released by the addition of SDS gel sample loading buffer. The released protein was then subjected to Coomassie staining and to a Western blot analysis using monoclonal antibody directed against MBP. Fig 5 panel A shows the original *E*. *coli* lysates (lanes 2 and 5), the lysates after incubation with the chitin beads (lanes 3 and 6), and the proteins bound to the chitin beads (lanes 4 and 7). Panel B shows a Western blot using the antibody directed against MBP for the same samples seen in panel A. As shown in Fig 5B, the MBP::LysM1 bound to the chitin magnetic beads (lane 4) while the control MBP did not (lane 7), indicating that LysM1 specifically binds to chitin. We found that MBP::LysM2 also bound to chitin (S6 Fig). Competition experiments in which the MBP::LysM1 was incubated with glucose, N-aceteylglucosamine, or with the major cell wall glucans, laminarin (β-1,3-glucan), and lichenin (β1-3/β1–4 mixed glucan) prior to the addition of the chitin magnetic beads, showed that the sugars and glucans were unable to compete with the chitin for the LysM1 carbohydrate binding. We conclude that LysM1 and LysM2 are chitin-binding proteins and associate with fungal cell walls by binding to cell wall chitin.

### LysM1 and LysM2 bind to glycoproteins from human skin lysates

**Fig 6:**
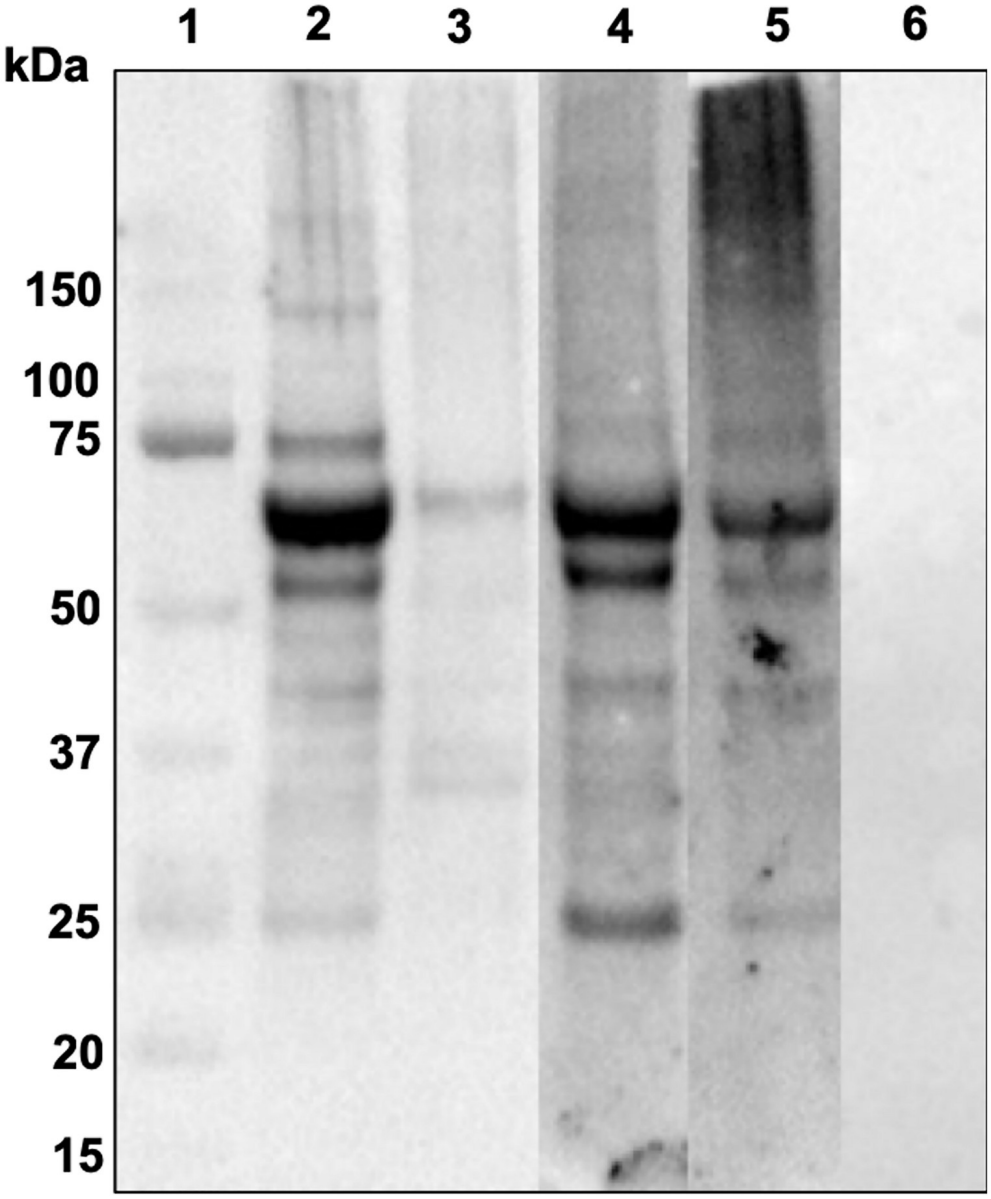
LysM1 and LysM2 bind to glycoproteins from human skin lysates. Proteins from human skin lysates were subjected to SDS PAGE, and the ability of MBP::LysM1, MBP::LysM1 domains 2 and 3, MBP::LysM2, and MBP to bind to these proteins was determined in a Western blot type assay with antibody directed against MBP. Lane 1 contains molecular weight markers. Lanes 2 through 6 contain human skin lysate (10 ugr). The lysate in lane 3 was treated with PNGase F prior to being subjected to electrophoresis. Lanes 2 and 3 were probed with MBP::LysM1. Lane 4 was probed with MBP::LysM1 domains 2 and 3. Lane 5 was probed with MBP::LysM2. Lane 6 was probed with MBP as a control. Note that LysM1, LysM1 domains 2 and 3, and LysM2 were able to bind to skin lysate proteins (lanes 2, 4, and 5) and the binding of LysM1 was largely abolished when the sample was treated with PNGase F to remove the N-linked oligosaccharides (lane 3).

With LysM1 being a multiple domain protein, we considered the possibility that it might bind to other substrates besides chitin. In particular, we considered that it might also recognize host proteins. To test this hypothesis, we carried out Western blot type experiments using human skin lysates and purified MBP::LysM1, MBP::LysM2, and the control MBP. The skin lysate proteins were subjected to SDS PAGE and transferred to membranes. The membranes were incubated with MBP::LysM1, MBP::LysM2, or MBP. Following a wash step, the membranes were incubated with monoclonal antibody directed against MBP and secondary antibody as described in Materials and Methods. As demonstrated in the Western blot experiment in Fig 6, MBP::LysM1 (lane 2) and MBP::LysM2 (lane 5) were able to bind to several proteins in the human skin lysate, while the MBP control (lane 6) did not.

Since LysM domains are carbohydrate-binding domains, we hypothesized that LysM1 and LysM2 might be recognizing some structure in the post-translation oligosaccharide modifications present on glycoproteins in the skin lysate. To determine if N-linked oligosaccharides might contain the binding site for LysM1, we asked if deglycosylation with PNGase F would abolish the binding of LysM1 to the skin lysate proteins. As shown in Fig 6 (lane 3), LysM1 binding to skin lysate proteins was largely abolished when the N-linked oligosaccharides were removed by PNGase F treatment. We conclude that LysM1 must recognize an element found on the N-linked oligosaccharides present on skin lysate glycoproteins. We hypothesize that the LysM1 may be binding to the chitobiose structure present at the base of the N-linked oligosaccharides.

### LysM1 domains 2 and 3 bind chitin and human skin glycoproteins

**Fig 7:**
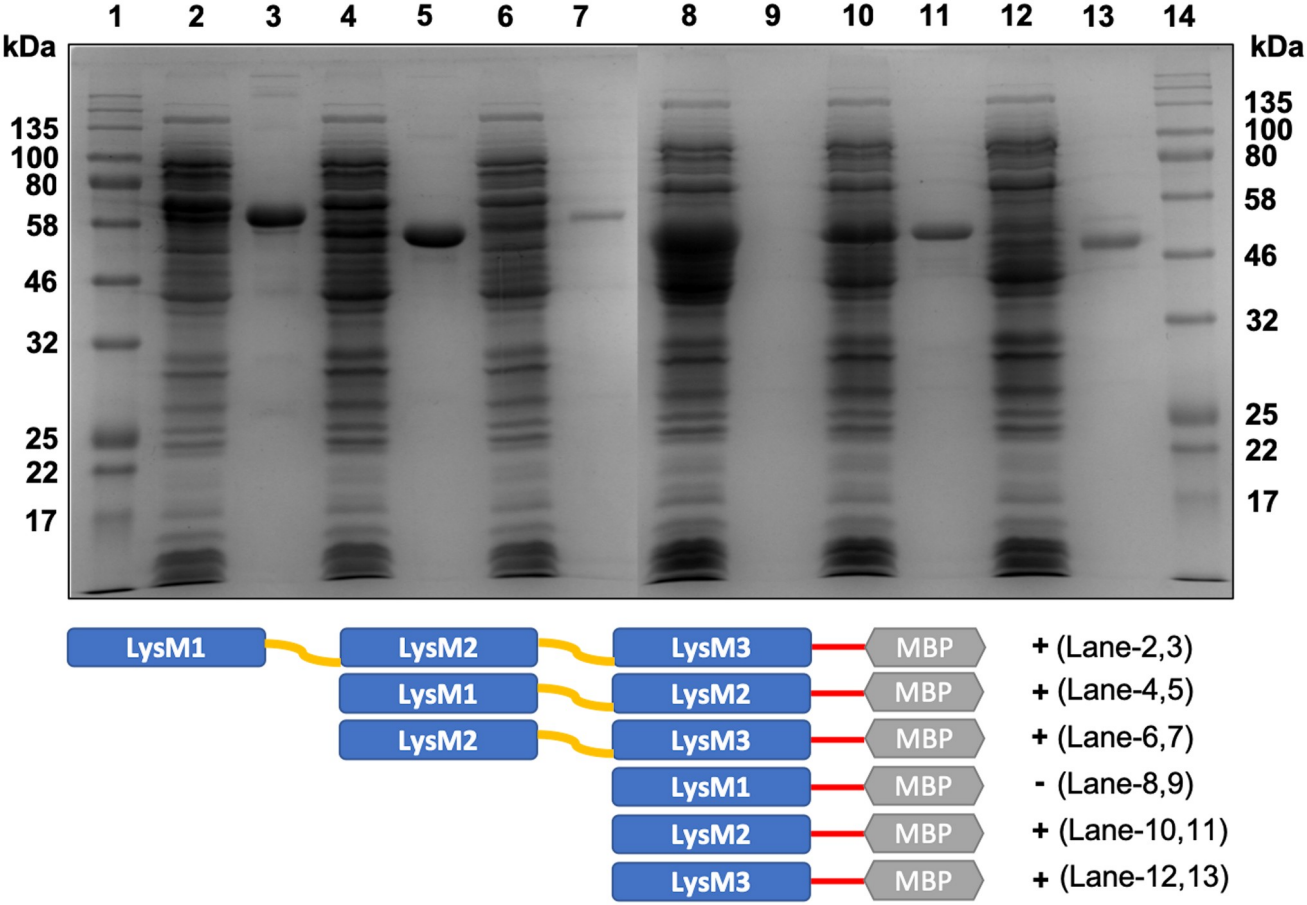
Chitin-binding activity of individual LysM1 domains. Constructs encoding chimeric MBP::LysM1 domains were expressed in *E*. *coli* and the various chimeric proteins were tested for chitin binding with the magnetic chitin beads. The figure shows a Coomassie blue stained SDS gel. Lanes 1 and 14 contain molecular weight markers. Lanes 2, 4, 6, 8, 10, and 12 show *E*. *coli* lysates containing full-length LysM1 (lane 2), LysM1 domains 1 and 2 (lane 4), LysM1 domains 2 and 3 (lane 6), LysM1 domain 1 (lane 8), LysM1 domain 2 (lane 10), and LysM1 domain 3 (lane 12). Lanes 3, 5, 7, 9, 11, and 13 show the proteins bound to chitin magnetic beads from lysates with full-length LysM1 (lane 3), LysM1 domains 1 and 2 (lane 5), LysM1 domains 2 and 3 (lane 7), LysM1 domain 1 (lane 9), LysM1 domain 2 (lane 11), and LysM1 domain 3 (lane 13). Note that MBP::domain 1 protein does not bind to the chitin magnetic beads (lane 9), while all of the other constructs are able to bind chitin. Below the figure is a graphic representation of the regions found in the different chimeric protein constructs.

As shown in Fig 1, LysM1 has three putative LysM domains, but the first of these is a truncated domain and is thus likely to be a nonfunctional domain, while the other two domains show high homology to each other and to other LysM proteins that bind to chitin (Fig 1). All three domains in LysM2 are full-length domains and show high homology to each other. We considered the possibility that different domains might be involved in binding chitin and human skin lysate proteins. To elucidate the binding specificities of the individual LysM domains in LysM1, a series of pMAL5-p5X plasmids were generated that contained the individual LysM domains as well as a plasmid that contained domains 1 and 2 and a plasmid that contained domains 2 and 3. These plasmids allowed us to express the individual LysM domains as chimeric MBP::LysM domain proteins in *E*. *coli*. Fig 7 shows that the individual LysM domain proteins are expressed in the *E*. *coli* lysates (shown in lanes 2, 4, 6, 8, 10, and 12). Chitin magnetic beads were used to determine if the individual LysM domains had chitin binding activity (lanes 3, 5, 7, 9, 11, and 13). As shown in Fig 7, all of the constructs containing either domain 2 or domain 3 gave positive binding results in the chitin magnetic bead assays. The constructs with domain #2 and with domain #3 bound to the chitin (lanes 11 and 13), but the construct with domain #1 did not bind (lane 9). These results indicate that domains 2 and 3 function as chitin binding domains, and that the truncated domain #1 does not bind chitin. We used the construct containing domain 2 and domain 3 in our assay for binding skin lysate glycoproteins (Fig 6, lane 4) and showed that these domains recognize the skin lysate glycoproteins. From our results, we conclude that domains 2 and 3 are responsible for the chitin binding activity and for the skin lysate glycoprotein N-linked oligosaccharides binding activity.

### LysM1 can also recognize N-linked oligosaccharides on *N*. *crassa* glycoproteins

**Fig 8:**
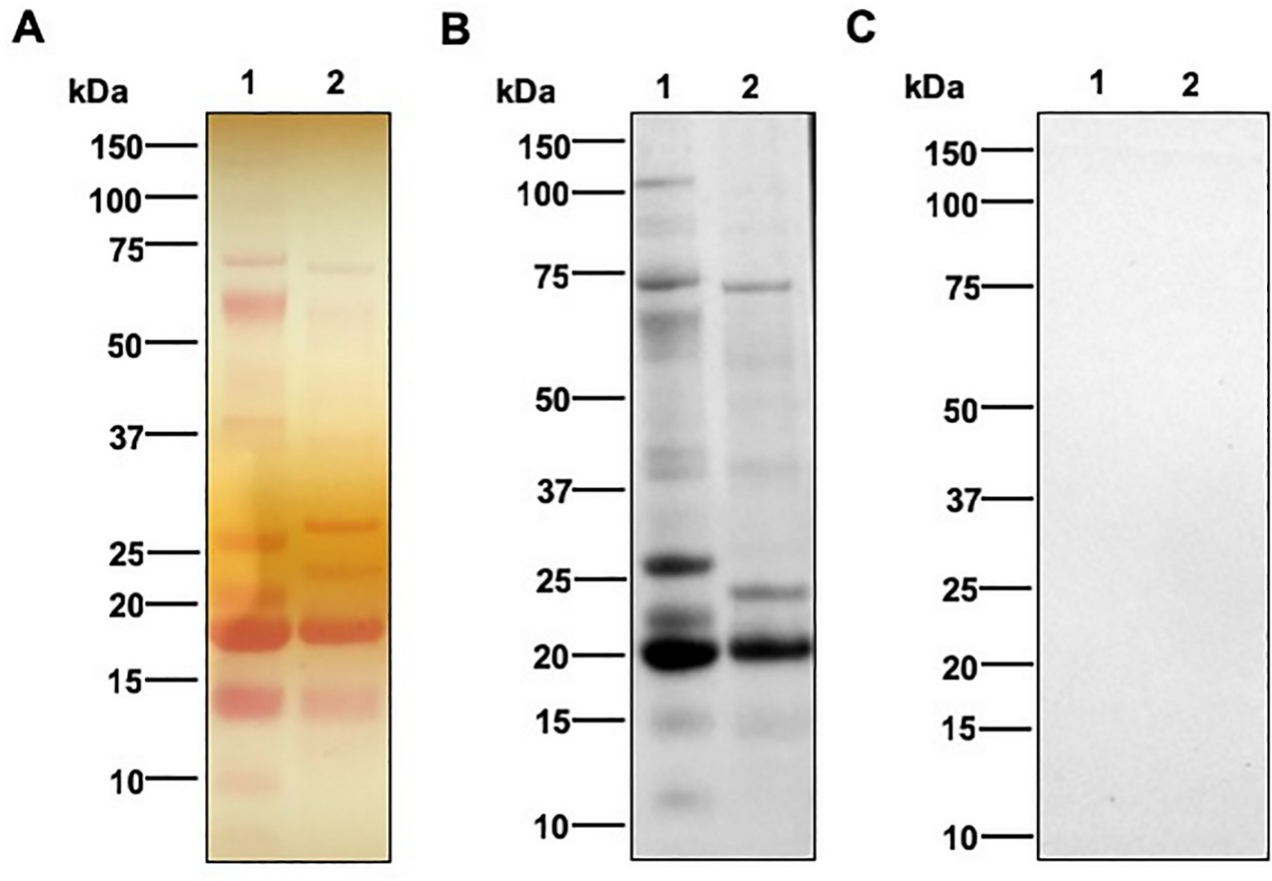
LysM1 binds to fungal cell wall glycoproteins. Cell wall glycoproteins released into the medium by a cell wall mutant were separated by SDS PAGE and used in Western blot type experiments. Panel A shows the secreted glycoproteins as assessed by Ponceau S staining of the nitrocellulose filter. Panel B shows the Western blot type experiment using MBP::LysM1. Panel C shows the Western blot type experiment using the control MBP. The cell wall glycoproteins in lane 2 of all three panels were treated with Endo H prior to being subjected to electrophoresis.

**Fig 9:**
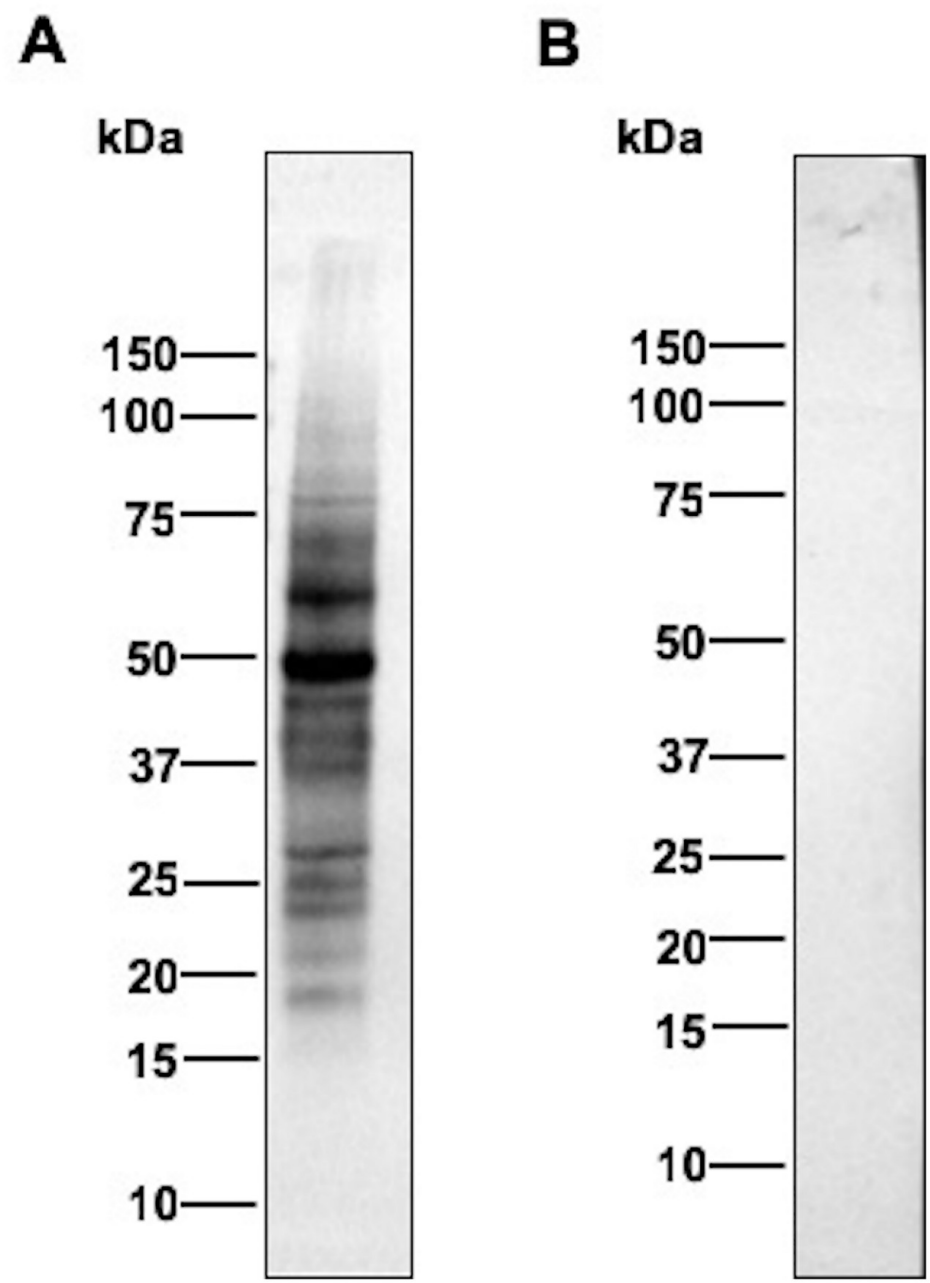
LysM1 is able to specifically bind to glycoproteins in *N*. *crassa* lysate. Proteins from wild type *N*. *crassa* were subjected to SDS PAGE and Western blot type experiments with either MBP::LysM1 (panel A) or control MBP (panel B).

To further examine the binding of LysM1 to N-linked oligosaccharides, we assessed whether LysM1 could bind to *N*. *crassa* glycoproteins. We have previously demonstrated that *N*. *crassa* glycoproteins, like the glycoproteins found in other fungi, contain high mannose type oligosaccharides with an unmodified chitobiose attached to asparagine in the context of an N-X-T or N-X-S amino acid sequence [32, 33]. The structure is modified by the addition of a galactomannan similar to the modification of yeast N-linked oligosaccharides with the outer chain mannan [32]. Fig 8 shows Western blot type experiments in which *N*. *crassa* cell wall glycoproteins were separated by SDS PAGE and the ability of the MBP::LysM1 to bind to the glycoproteins was assessed. Panel A shows the staining of the c ell wall glycoproteins on the membrane with Ponceau S stain. Panel B shows the binding of the MBP::LysM protein to the secreted glycoproteins in a Western blot type experiment. Panel C shows the control Western blot type experiment with MBP. As shown in Fig 8B lane 1, MBP::LysM1 was able to bind to the glycoproteins, while the MBP control does not (Fig 8C lane1). When the glycoproteins were treated with Endo H, which removes some of the N-linked oligosaccharides (Fig 8B, lane 2), the sizes of the glycoproteins were reduced and the LysM1 binding was diminished. Fig 9 shows an experiment in which *N*. *crassa* cellular lysate was subjected to the Western blot type analysis with MBP::LysM1 and control MBP. The control MBP is unable to recognize any proteins in the lysate (panel B), while MBP::LysM1 is able to recognize a number of glycoproteins (panel A). We conclude that LysM1 can recognize the N-linked oligosaccharides present on fungal glycoproteins, as well as recognizing the N-linked oligosaccharides on human skin glycoproteins. The chitobiose at the base of the N-linked oligosaccharide, is the most likely candidate for LysM1 binding. While human and fungal glycoproteins differ in that extracellular human glycoproteins have largely complex or hybrid type N-linked oligosaccharides and fungi have high mannose type N-linked oligosaccharides, the chitobiose present at the base of the structure is a common element found on both types of glycoproteins.

## Discussion

LysM proteins are carbohydrate-binding proteins that have been found in bacteriophages, bacteria, fungi, plants, and animals. LysM proteins have been shown to bind to N-acetylglucosamine-containing oligosaccharides, including bacterial cell wall peptidoglycans and chitin. LysM motifs are found in a variety of proteins and are thought to function in allowing these proteins to specifically recognize and bind to glycans. The recent sequencing of the *T*. *rubrum* genome showed that the genome contained fifteen LysM domain proteins, while related nonpathogens contained between zero and four such proteins [28]. The increase in LysM proteins encoded in the *T*. *rubrum* genome suggests that these proteins may be important for the pathology of the fungus. Three of these proteins had a LysM domain and a chitinase domain, suggesting the encoded proteins functioned as chitinases. Six of the LysM domain proteins contained multiple LysM domains, and no obvious homologies to other proteins.

The focus of our research efforts was to identify what glycans the *T*. *rubrum* LysM proteins recognize and to determine how they might contribute to the pathology of the fungus. We picked two of the multiple LysM domain proteins, LysM1 (encoded by gene TERG_05627) and LysM2 (encoded by gene TERG_01873) for our studies. Codon-optimized versions of these two proteins were designed for expression in *N*. *crassa*, a model filamentous fungus, with the intent of making the proteins with their normal post-translational modifications. The two proteins were produced as chimeric LysM1::GFP and LysM2::GFP proteins to allow us to follow the expression and determine the location of the expressed proteins. Expression of these proteins in *N*. *crassa* demonstrated that the proteins were targeted to the cell wall (Fig 2). To further characterize the proteins, we produced both proteins in *E*. *coli* as chimeric maltose binding proteins, MBP::LysM1 and MBP::LysM2. We were able to demonstrate that the *E*. *coli*-produced LysM1 and LysM2 were able to bind to the cell walls of both *T*. *rubrum* and *N*. *crassa* (Fig 4). This demonstrated that the *E*. *coli*-produced chimeric proteins were functional and that the fungal post-translational modifications are not required for their binding activity. We directly asked if LysM1 and LysM2 were chitin-binding proteins by using a magnetic chitin-bead assay system and found that both proteins were able to bind to chitin polymers (Fig 5).

We also asked whether the *E*. *coli*-produced LysM1 and LysM2 proteins could recognize any proteins in human skin lysates. In a Western blot type of experiment, we found that both LysM1 and LysM2 were able to recognize proteins in a human skin lysate (Fig 6). An experiment in which the skin lysate proteins were treated with PNGase F to remove the N-linked oligosaccharides demonstrated that the N-linked oligosaccharides were needed for the recognition of the proteins by LysM1 and LysM2 (Fig 6). These results suggest that LysM1 and LysM2 are recognizing the N-linked oligosaccharides present on skin lysate glycoproteins.

We also tested the ability of our recombinant LysM1 proteins to bind to fungal glycoproteins, and demonstrated that LysM1 could bind to *N*. *crassa* cell wall glycoproteins (Fig 8) and glycoproteins in a cellular lysate (Fig 9). We further showed that the binding to cell wall proteins was sensitive to Endo H glycosidase treatment, demonstrating that the fungal N-linked oligosaccharides were being recognized by LysM1. This provides strong support for the hypothesis that the chitobiose found at the base of the N-linked oligosaccharides, a feature of the N-linked oligosaccharides that is found on both fungal and human glycoproteins, is being recognized by the *T*. *rubrum* LysM proteins.

We tested the ability of each of the LysM domains in LysM1 to bind to chitin and to skin lysate glycoproteins. The second and third domains were able to bind to both chitin and skin lysate glycoproteins (Figs 6 and 7). We concluded that all of the binding activity of LysM1 is found in the second and third LysM1 domains and that these domains recognize both of the substrates, chitin and skin lysate glycoprotein.

To the best of our knowledge, this is the first report of a LysM protein binding to N-linked oligosaccharides as well as binding to chitin. LysM domains have been previously shown to have the ability to bind both peptidoglycan and chitin [15, 17], so there is a precedence for having two N-acetylglucosamine-containing substrates being recognized by a LysM domain. Studies on the structures of LysM proteins demonstrate that the LysM domain has a shallow groove capable of accommodating N-acetylglucosamines [14, 16, 34]. The LysM domain found in a *Magnaporthe oryzae* CVNH-LysM protein is able to bind the N-acetylglucosamine-N-acetylmuramic acid disaccharide and the chitin trisaccharide [34]. A LysM protein from a plant chitinase was shown to be able to bind chitotriose [16]. If the binding of LysM1 and LysM2 to the skin lysate glycoproteins is through the chitobiose at the base of the N-linked oligosaccharide, this would represent binding with only two N-acetylglucosamine residues present in the binding groove, a situation similar to that found in the binding of the N-acetylglucosamine-N-acetylmuramic acid to the *M*. *oryzae* CVNH-LysM protein.

One important consideration in evaluating the function of the LysM proteins is that different members of the LysM family may be expressed at different times in the infection process or in different cell types. Although the amplification of the LysM genes in the *T rubrum* genome suggests that the proteins have a role in host infection, detailed expression data on the individual genes during infection is not currently available. It is possible that different LysM proteins are expressed in the spores, during the initiation of infection, and in an established infection.

Our results suggest that the *T*. *rubrum* LysM proteins could serve three important functions during the infection process. First, the LysM proteins could bind to the *T*. *rubrum* cell wall chitin and shield cell wall components (chitin and perhaps glucans and mannans) from the human innate immune system. The role of chitin in the human innate immune system has not been firmly established, but Elieh Ali Komi et al recently reported that chitin functions as a PAMP (Pathogen-associated molecular pattern) for the human innate immune system [7]. By binding to the cell wall chitin, the LysM proteins could help the fungus evade the human innate immune system. The LysM proteins could protect the cell wall chitin from host chitinases which release chitin fragments to activate inflammation [7]. The proteins could also bind to released chitin fragments and thereby provide protection from the host immune system. LysM proteins produced by plant pathogenic fungi have been shown to serve both of these types of functions to allow plant pathogens to evade recognition by their hosts [22–27, 35]. In a similar manner, the *T*. *rubrum* LysM1 and LysM2 could provide the fungus with the ability to evade chitin recognition by the human immune system. The binding of LysM proteins to the *T*. *rubrum* cell wall could also help shield cell wall glucans and mannans from the innate immune system.

A second possible function that the LysM proteins could provide during *T*. *rubrum* infections would be to bind to the N-linked oligosaccharides present on the *T*. *rubrum* cell wall and secreted glycoproteins. By binding to these glycoproteins, the pathogen may be able to shield these proteins from the human immune system.

The third and perhaps most interesting possible role for LysM proteins would be to facilitate adhesion to the human skin. LysM proteins with multiple LysM domains could simultaneously bind to fungal cell wall chitin or cell wall glycoproteins and to skin glycoproteins, and thereby provide adhesion between the fungal cell wall and skin glycoproteins. We don’t have binding affinity measurements for the LysM domains in LysM1 or LysM2 binding to chitin or to skin lysate N-linked oligosaccharides to assess how tightly the proteins bind to the two different substrates. We would anticipate that host glycoproteins, where we envision two N-acetylglucosamine residues binding to the LysM protein, will have a much lower affinity than the longer chitin oligosaccharide. However, if the fungus produces an excess of LysM proteins containing multiple LysM domains over the amount of cell wall chitin, the excess LysM domains would be available to bind skin glycoproteins and thereby facilitate adhesion to the skin. With a high concentration of LysM proteins, the *T*. *rubrum* cell wall would have “high avidity” for the host skin and could effectively facilitate adhesion to the skin. In effect, the fungus cell wall LysM proteins would represent a “Velcro coat” capable of binding to human skin.

## Supporting information

S1 Table Primers used to clone LysM1, LysM1 domains, and LysM2 sequences into the pMAL-p5X vector. (DOCX)

S1 Fig Complete sequence of pKB01. (DOCX)

S2 Fig Complete sequence of pKB02. (DOCX)

S3 Fig LysM1::GFP and LysM2::GFP expression in the *N*. *crassa Δoch-1* strain. Cells from a transformant expressing LysM1::GFP (top row), from a transformant expressing LysM2::GFP (middle row), and from the non-transformed parental strain (bottom row) were examined by confocal microscopy. The left panels show the GFP images, the middle panels show the DIC images, and the right panels show the merged images.
(TIFF)

S4 Fig Purification of the MBP::LysM2 protein. Lane 1 contains molecular weight markers and lane 2 contains the amylose resin purified chimeric MBP::LysM2 protein.
(TIFF)

S5 Fig LysM1 binds to *N*. *crassa* cell walls. *N*. *crassa* hyphae were incubated with purified MBP::LysM1 and the binding of LysM1 to the cells was visualized with a fluorescent antibody protocol using antibody directed against MBP. The top row contains cells stained for the binding of MBP:: LysM1 and the bottom row contains cells strained for the binding of the MBP control. The left panels show the fluorescent images. The middle panels show the DIC images. The right panels contain the merged images.
(TIFF)

S6 Fig LysM2 binds to chitin. Lane 1 contains molecular weight markers. Lane 2 contains *E coli* lysate from a transformant expressing MBP::LysM2. Lane 3 contains the chitin binding proteins from the transformant lysate.
(TIFF)
