# Supplementary material for: Trichophyton rubrum LysM proteins bind to fungal cell wall chitin and to the N-linked oligosaccharides present on human skin glycoproteins
Source: PLoS One. 2019 Apr 4;14(4):e0215034. doi: 10.1371/journal.pone.0215034 (PMC6449025; doi:10.1371/journal.pone.0215034)
Supplement: S1 Table — (DOCX) [file pone.0215034.s001.docx]

**S1 Table. Primers used to clone LysM1, LysM1 domains, and LysM2 sequences into the pMAL-p5X vector.** Primers TERG_05627F and TERG_05627R were used for cloning full-length LysM1. Primers TERG_05627F and 5627Do.1R were used for cloning LysM1 domain 1. Primers 5627Do.2F and 5627Do.2R were used for cloning LysM1 domain 2. Primers 5627Do.3F and TERG_05627R were used for cloning LysM1 domain 3. Primers TERG_05627F and 5627Do.2R were used for cloning LysM1 domains 1 and 2. Primers 5627Do.2F and TERG_05627 were used for cloning LysM1 domains 2 and 3. The TERG_01873F and TERG_01873R primers were used for cloning full-length LysM2. The NdeI and BamH1 sites used for inserting the PCR products into the vector are shown in bold type.

| Sequence name | Sequence |
| --- | --- |
| TERG_05627F | CATA**CATATG**CGCGTCCTCCACCCCCGTGCTGTG |
| 5627Do.1R | CTGC**GGATCC**TCAAGCGGTGCTGGTGGTGGTGG |
| 5627Do.2F | CATA**CATATG**GCCAAGCCCACCATGACCCCCACC |
| 5627Do.2R | CTGC**GGATCC**TCAGGGGGTGGGCATGGGCATGG |
| 5627Do.3F | CATA**CATATG**ATCTCTACCTCCATGCCCATGCCCACCC |
| TERG_05627R | CTGC**GGATCC**TCAGACGCCGATGCAGACGTAGTAACC |
|  |  |
| TERG_01873F | CAGC**CATATG**AAGAAGCCGAACGGTCCCACGTACGC |
| TERG_01873R | CTAA**GGATCC**TCACTTGCCGCCGACGCAGACGTAGTAG |
