# Supplementary material for: Trichophyton rubrum LysM proteins bind to fungal cell wall chitin and to the N-linked oligosaccharides present on human skin glycoproteins
Source: PLoS One. 2019 Apr 4;14(4):e0215034. doi: 10.1371/journal.pone.0215034 (PMC6449025; doi:10.1371/journal.pone.0215034)
Supplement: S2 Fig — (DOCX) [file pone.0215034.s003.docx]

S2 Fig. Complete sequence of pKB02 for the expression of a chimeric LysM2::GFP protein in *Neurospora crassa*. LysM2 sequences are in blue and GFP sequences are in green.

TATAGTGTCACCTAAATCGTATGTGTATGATACATAAGGTTATGTATTAATTGTAGCCGCGTTCTAACGACAATATGTCCATATGGTGCACTCTCAGTACAATCTGCTCTGATGCCGCATAGTTAAGCCAGCCCCGACACCCGCCAACACCCGCTGACGCGCCCTGACGGGCTTGTCTGCTCCCGGCATCCGCTTACAGACAAGCTGTGACCGTCTCCGGGAGCTGCATGTGTCAGAGGTTTTCACCGTCATCACCGAAACGCGCGAGACGAAAGGGCCTCGTGATACGCCTATTTTTATAGGTTAATGTCATGATAATAATGGTTTCTTAGACGTCAGGTGGCACTTTTCGGGGAAATGTGCGCGGAACCCCTATTTGTTTATTTTTCTAAATACATTCAAATATGTATCCGCTCATGAGACAATAACCCTGATAAATGCTTCAATAATATTGAAAAAGGAAGAGT

ATGAGTATTCAACATTTCCGTGTCGCCCTTATTCCCTTTTTTGCGGCATTTTGCCTTCCTGTTTTTGCTCACCCAGAAACGCTGGTGAAAGTAAAAGATGCTGAAGATCAGTTGGGTGCACGAGTGGGTTACATCGAACTGGATCTCAACAGCGGTAAGATCCTTGAGAGTTTTCGCCCCGAAGAACGTTTTCCAATGATGAGCACTTTTAAAGTTCTGCTATGTGGCGCGGTATTATCCCGTATTGACGCCGGGCAAGAGCAACTCGGTCGCCGCATACACTATTCTCAGAATGACTTGGTTGAGTACTCACCAGTCACAGAAAAGCATCTTACGGATGGCATGACAGTAAGAGAATTATGCAGTGCTGCCATAACCATGAGTGATAACACTGCGGCCAACTTACTTCTGACAACGATCGGAGGACCGAAGGAGCTAACCGCTTTTTTGCACAACATGGGGGATCATGTAACTCGCCTTGATCGTTGGGAACCGGAGCTGAATGAAGCCATACCAAACGACGAGCGTGACACCACGATGCCTGTAGCAATGGCAACAACGTTGCGCAAACTATTAACTGGCGAACTACTTACTCTAGCTTCCCGGCAACAATTAATAGACTGGATGGAGGCGGATAAAGTTGCAGGACCACTTCTGCGCTCGGCCCTTCCGGCTGGCTGGTTTATTGCTGATAAATCTGGAGCCGGTGAGCGTGGGTCTCGCGGTATCATTGCAGCACTGGGGCCAGATGGTAAGCCCTCCCGTATCGTAGTTATCTACACGACGGGGAGTCAGGCAACTATGGATGAACGAAATAGACAGATCGCTGAGATAGGTGCCTCACTGATTAAGCATTGGTAA

CTGTCAGACCAAGTTTACTCATATATACTTTAGATTGATTTAAAACTTCATTTTTAATTTAAAAGGATCTAGGTGAAGATCCTTTTTGATAATCTCATGACCAAAATCCCTTAACGTGAGTTTTCGTTCCACTGAGCGTCAGACCCCGTAGAAAAGATCAAAGGATCTTCTTGAGATCCTTTTTTTCTGCGCGTAATCTGCTGCTTGCAAACAAAAAAACCACCGCTACCAGCGGTGGTTTGTTTGCCGGATCAAGAGCTACCAACTCTTTTTCCGAAGGTAACTGGCTTCAGCAGAGCGCAGATACCAAATACTGTCCTTCTAGTGTAGCCGTAGTTAGGCCACCACTTCAAGAACTCTGTAGCACCGCCTACATACCTCGCTCTGCTAATCCTGTTACCAGTGGCTGCTGCCAGTGGCGATAAGTCGTGTCTTACCGGGTTGGACTCAAGACGATAGTTACCGGATAAGGCGCAGCGGTCGGGCTGAACGGGGGGTTCGTGCACACAGCCCAGCTTGGAGCGAACGACCTACACCGAACTGAGATACCTACAGCGTGAGCATTGAGAAAGCGCCACGCTTCCCGAAGGGAGAAAGGCGGACAGGTATCCGGTAAGCGGCAGGGTCGGAACAGGAGAGCGCACGAGGGAGCTTCCAGGGGGAAACGCCTGGTATCTTTATAGTCCTGTCGGGTTTCGCCACCTCTGACTTGAGCGTCGATTTTTGTGATGCTCGTCAGGGGGGCGGAGCCTATGGAAAAACGCCAGCAACGCGGCCTTTTTACGGTTCCTGGCCTTTTGCTGGCCTTTTGCTCACATGTTCTTTCCTGCGTTATCCCCTGATTCTGTGGATAACCGTATTACCGCCTTTGAGTGAGCTGATACCGCTCGCCGCAGCCGAACGACCGAGCGCAGCGAGTCAGTGAGCGAGGAAGCGGAAGAGCGCCCAATACGCAAACCGCCTCTCCCCGCGCGTTGGCCGATTCATTAATGCAGGTTAACCTGGCTTATCGAAAT

TAATACGACTCACTATAGGGAGACCGGCAGATCC

AATGCGGATGGATTCGCTCACTCGGTGCCGCGGCGGTGCGAAGTTCCTGCCGTGGATTGGGGGATTGCACAGACGGTGGAGTGCTTGCTACAGCTAGGTACCCAGTTGCACACCAAACGGTGCCCCTGTGCCCCTGCGTCTAGGTGTGTCCTGTGAGCAACTGCGCCCGCGCGCGGCACCGGGAAGCTGCGGGAACCTCAATGGATGATTGGCGCCAGATCCCCGGCATCACCTCAGCACATGGGCAGCCCGAGAAGGAATCGAGGGTAGAAGATATTCCAGGGTTTCCGCTAGCACAACTTCCAAGCGCCTCTACCGCTACTCCACACACCCACGCTGAATGGCACAACCACTGTAGGCCTAGCGGTCCCAGCTCCCCGTAACCACGCAGCCCCAGCGACCGAGGTCATCATGACACTATGACGACAATCTGGCGGGAGACTTGGGAAAATCGATGACGGGCACCTACCTTGGGAACAATAGAGCAAGTCCAGTCAACGCCCTAGGGAAGGGGGAAAAAAAAAAAGAGCCCATTCTCACAAGGAGCAACACTGTGCTGCTATCATAGTACACTACAAGCAAAGTCGGGATGTTGCTAGAATCCGTCGGCTACGTCGGTAGATACCTCCCATAGGCAGTAAGTCTAGGTATCCAATTTATAGTTCCGCGTATTTCTTCGTCGAACGGGGCACAGCAAAGCCTAACAGCCGCAGTGCCTCGCAACCAACACCAAGCAGTAATCTCATCCATCCATACAGATCCTCCCACAATATCTTTACATGCGCACTTGCGCTGTGTTAGCAGTTTAGGTTCCACTACCAAGGCTGACACGAGCAGCACTGACAGCAAACTGCTGCTTGGGGCCAAGAAATTGGCAGTTTGCTGAGCACCTACATATCAGTACACATCAACCCTGGTTTAATTGTTAGCCTGGTACTGTTAACCGTCATGAACGAGGACGTGGAGGAAGAACCACGCTAACAATTGAACCAGGGTTGATGTATACCTCTCTTACCATATTTGCTTGCTTCCACGTCCCTATCCTCAAGCGATTTTGTTCTTGACTTTTTGTATTTCAACGCAAAGGCCATTCAGGTTTCTCATGTTCAACCCTTTGGATGGCGATGCCGTTCGTCCCAGGACCTCGTGACCTTTGAAAGACGAGAAATATATCCCAAAGCAACTTCTGCTTATGGACAAGCAACTGCATGGCGGCCCACAACTCATATCTCGCGTAATCATCCAAACGCTAGGCCCGAGTACTCTACTGTTCTCGAGACACCTACCATAATATGTACCCAATCAGACATGACAACAGTCAACCAAACATACCCTGATCCGGCCAAACCCTGACATCCCTAGATGATGGTTAGGCACACAAATGGAAAACGGGACTCGTTGGTTAGGTGGGAACGCTTGTATTGCAGCCAGCCAACCGACTCTCCTCGTCCCTCTCGAGTCCCGTTATTGCCGTTTGACCCAGCCGCCCAGCCGCCAAACAGCTGAGGGAGCCAATGGTCTAATACACCGTCCAACGGCCCCAGTGATGTTTCAAACTTGATTGACAGCGAACGAAACCCCTGAAACCCGTTATAGATATGTATAGATTTGACGGGGTAGCTTGGCCCT

TAATACGACTCACTATAGGGCGAATTGGAGCTCCACCGCGGTGGCGGCCGC

TAGAAGGAGCAGTCCATCTGCGTGAATCACGAGAGAATCAGCTACTTTGAATCGATGGATGCAGCTACCAGAAGTCACTCAGTTCGTTCAAAGCCACATCACTGGGCACTTCCATTGGGACAGGCATTGATCGGACGAGACCGACTTCTGGCCGCTTTCAACAGCCACATTATATCCATGTCACGGCTACGCGTCGGCCTTCGGTAACAGAAAAGCACACAGACAGCGATTGTGACATGGATTCGGGCAAAGCATTGGTGGTCGCACCAGGTCATCCTGAGTGTGCAGTGGCTGCTATTCAGATTTCATCTAACTGCGGGAGAGGGGTTCAAAGGGGCGTGACGTCACAGACAACGGGTGAAGGACGAAGATTGCCTCACTTCTTTGCTAGCAATTTGCCTGCAAAGAAGGCGCACATGACAAGCAAACAACTGGGAAACCACTATTGAATACCCACAATGCAAAGCTCGGAAGGTACGTCTTGATTGCAGTGTGTCGAGTGTCAAAAAAGAAGCAAGTGTTCATGCAAGCCAAAATTGGCACCTCCTCCACTTCTCCGAGTGCCCCACCCGAACCTCCAGGCGAGATGGCCGGAACATACCATCCGCGTTGGGATTATGACGTATCTCCTTCTTCTTCACATGATTCCATCCCGTTGTTGCTTGTTTGCGAGCTGTGACGGGAGATCGTAGATGCCACTTCGGGCCAGGCAGGCAGTGCAGGCAGCCAGGAACACAAGCTTCCAACTTGGTCATCTCGATTGCCGATTAAGGGAACCAAATGCCTATATAAGACTGTCCTCCCACCTCCCCAATACCATTCTTTTCTTCTTCCATCATCAGCCAACAAAGCAATCACATCTTCACTACTTCAAATCAACACAACACTCAAACCACTTTCACAACCCCTCACATCAACCAAA

CTCTAGAACTAGTCACCAAATGAAGATCTCCAGCCTCAGCATCCTCCCCCTCCTCGGTGTTGTCTCCGCTGGCATCCACGGCAAGAAGCCGAACGGTCCCACGTACGCAGGTACCAACCCCGGCTGCACCTGGTACCTCGACCTCGTCGATGATAGCTACACCTGCGAGAACATCGAGAGCCAGTGGGATTTGAGCCACGAAGCCTTCGTGGCCTGGAACCCCGGTGTCAAGAAGGACTGCAGCGGGCTCAAGGTCGGTCTCTCCGTCTGCGTGGAGGCCCCCATGGAGTCCATCACCGCCACCCCCACGTCCGAGGCCTCCACGTCCTCGGAGACCTCTAGCGCCTCGCCCACCGCTTCGAGGCCCCCCTTGCCCTCTCCCACCCAGGATGGTTTGGTGAGCAACTGCACCAAGTTCCACCAGGCTGTCAGCGGTGACACCTGCAGCAAGATTATCTCCCGGTACAAGCCCATCACCCTCGACCAGTTCATCGAGTGGAACCCCGCTCTGGAGAAGGACTGCTCGGGGCTCTGGTCCGGTTACTACTACTGCGTCGGTATCCCCGGCACCCCCAGCGCGCCCCTCACGAGCATGCCGGCTAGCACGGCGCACAGCGGTACCAACTCCAACGCCACCGTCAAGCCCGCCGCCCCCGGCCCCACCCAGGATGGTATCGCCAACAACTGCCAGCGGTGGCACAAGGCGGTCTCGGGGAACACCTGCGCTAGCCTCCTCAAGCAGTACGGCACCTTCACCCTGGAGGAGTTCATCAAGTGGAACCCCGCCGTCGGCGAGGACTGCTCCGGCCTCTGGCTCAACTACTACTACTGCATCGGCATCCCCGGTACCCCCACCACGAAGAACTCCGTGCCCAAGCCCACCACCACCGCCTGCAACACCCCCGGTCTCCCCTCCCCCACCCAGCCGGGCGCTATCTGCCAGTGCAGCAAGTGGCACCAGGTCAGGCAGGGCGAGACCTGCGACAGCATTACCCGCAACTACCGCATTTCCATCTCCAAGTTCAAGGAGTGGAACCCCAACGTTGGCAAGGACTGCTACGGTCTCTGGCTCCGCTACTACGTCTGCGTCGGCGGCAAGTGGATCCCCGGGTTAATTAAC

ATGGTGAGCAAGGGCGAGGAGCTGTTCACCGGGGTGGTGCCCATCCTGGTCGAGCTGGACGGCGACGTAAACGGCCACAAGTTCAGCGTGTCCGGCGAGGGCGAGGGCGATGCCACCTACGGCAAGCTGACCCTGAAGTTCATCTGCACCACCGGCAAGCTGCCCGTGCCCTGGCCCACCCTCGTGACCACCTTCACCTACGGCGTGCAGTGCTTCAGCCGCTACCCCGACCACATGAAGCAGCACGACTTCTTCAAGTCCGCCATGCCCGAAGGCTACGTCCAGGAGCGCACCATCTTCTTCAAGGACGACGGCAACTACAAGACCCGCGCCGAGGTGAAGTTCGAGGGCGACACCCTGGTGAACCGCATCGAGCTGAAGGGCATCGACTTCAAGGAGGACGGCAACATCCTGGGGCACAAGCTGGAGTACAACTACAACAGCCACAACGTCTATATCATGGCCGACAAGCAGAAGAACGGCATCAAGGTGAACTTCAAGATCCGCCACAACATCGAGGACGGCAGCGTGCAGCTCGCCGACCACTACCAGCAGAACACCCCCATCGGCGACGGCCCCGTGCTGCTGCCCGACAACCACTACCTGAGCACCCAGTCCGCCCTGAGCAAAGACCCCAACGAGAAGCGCGATCACATGGTCCTGCTGGAGTTCGTGACCGCCGCCGGGATCACTCTCGGCATGGACGAGCTGTACAAGTAAGAATTCGATATCAAGCTTATCGATACCGTCGACCTCGAGGGGGGGCCCGGTACCCAGCTTTTGTTCCCTTTAGTGAGGGTTAATT

AGGGCGTGGACGGCTAATGGGGTCTGAATGCTAAAAGACACCATTTCCCACACTCCCTCTGATTTTTTTTATGGTTTTTTTTTATCTTCATTTTTCGCCGTCTTTTATTCGGTTGTTTGGGAGGTTCACATGCAACGTGCAAGACTGCAAGTTTTTTGAGATGGGCGCTTCATGCAATGCAGCATCACTGCAGGGACATTGTGACATCCACGTTTCGATCGTCTGTGCTTCCCCCGTTCATGTACGCATGATCTGAAAAAAAAAAAAAAAAAAAAGGTTGATGTAATAATGAAAATAAACACCCAGGGACGAGCACCTCATCTGCTGGAATAGGGAGAAAAACTCAAGGGCCGTTTGGGTCTCCACACTTACCAACAGATTCATGTACTGATCTTCAGCCTGTACAGAACAGTACAATGTCCAAAACTCGTTATCATTACTCTATGTGCCCAAAATGACCCAGATATACATCAACCCACTACATCCTTTTGTTCTTGTTTTTTTTTTTTTTTTTTGCTACTTCGAAAAGAATTTCCGTTTAGTTGCTCTTGCTCATGTGCTCAAGACGGATGCTGACCGCCCTTCTGTGAGCCTCGAGCTCCTCAACCTTAGCCAACTGCATGACAGCCTGGCCGACGTTTTTGAGACCCTCGGCAGTCAAGTTGGAGCTGGTAATGTGCTTGACGAACGAGGCGAAATTGACGCCAGAGTACTGCTTGCCAAAGCCATAGGTAGCTATGTGTCTTGACGTTAGCAGAAGAATCTGTGAAGCGGGGCTATTTATGATATGTAACTTACGCAGCGAGTGGTTAACACCAGCAGAGTAATCGCCAACGGACTCAGGAGTCCAGGCGCCAATGAAGACACTACCGGCGTTCATGACAAGATCGACAGCCTTCTCGGCCTCCTTGATCTGGAGGATCAAGTGCTCAGGAGCGTACTTGTTGCTGAGCTCCATGGCCTCCTCGACGGTCTTGACCTGCACGGTGATCGAGTGGGCGATGGAGCCACGGACAATCTGGACGCGAGGAAGCTCCGTAGCCTGACGGTGAACCTCGTCCTCAATAGCCTGAAGATGCTCCTCGTCGAGGTCAATAGCGATCAGGATGACCTGACTGTCAACGCCGTGCTCAGCCTGGGACAGGAGATCCGAGGCAACGAACGCGGGGTTGGCGTCCTTGTCAGCGATGACCAGCACCTCGGACGGGCCAGCGGGCATGTCAATACCAACGGCAGCGTTGGTGTCGTTGCTGACGAACATCTTGGCAGCAGTGACGAACTGGTTACCGGGGCCGAGAATCTTGTCGACCTTGGTGATGCTCTCGGTGCCGTAGGCCATGGCAGCTACGGCCTGGGCACCGCCGGCAAGCACGATGGACTCGGCCCCAACCTTGTGAGCGACGTGGACAATCTCGGGAGTGATGGTTCCGTCGGCGCGGGGAGGAGAGGCGAACACAATCTTGTTGCAGCCGGCGACCATGGCGGGAACACCCAGCATAAGGGCAGTGCTGGGGAGAACGGCGGTACCGCCGGGGATGTAGCAGCCGACGGCCTCGATGGGACGAGAGAAACGGCTGCAGACAACACCGGGCATGGTCTCGACCTGGAGGGGCTTCTCCTCCTTCTGGGCGGCGTGGAACTTGCGGATGTTCTCGAAGGACACGTCGATGGCAGCAATGGTCTCCTCAGGGAGCTGCATAAGCTCCTTGGGGAAGGGCGCCTTCAGGACGGGGCTAGTAAGAGAGGTAGCCTTCTCGAACTTGTGAGTGTACGACAGAACAGCCTTGTCGCCGTTCTTGCGGACGTCCTCGATGATGGGGACAATGATCTTGTAGATGGCATCGGACGACTTTTGCGCAGGACGCTTGAGAGCAGCATCGAGCTCCTCGGTAGAGACCTTGGAGGCGTCGAAACGTCTCATGGTGATCTTCTCAGGGGTGGTCTCCTGGGCGGCCTCCTTGGTGACAGGGGCCGAAGTGGCAGCGAGAGCGGACGCCGCAGGCTTGATGCCCTCCTTCTCAGCCCACTTACCCTTAGCATCTCCAGTCCTGCGCTTGACCTTCCAGCTCTTGGCGTCAAGGCTCCTTTCGATATCGGCAAGAGTAACGCCGGCAGCAACGGCCCTGGTAAGAGCAAAGTAGAAGAGATCGGCAGCCTCAAAGGCGATTTCCTGGGGGGTCTGAGCGGTGCAGAGCTCCTCAGCCTCCTCCATGATCTTGGCCCGGACTAGCTTCTCATCGGAGAAGAGACGGGCAGTGTAGGAGCCCTCGGGGGCAGACTGTTTCCTCGAAATCAAAGTCTGCTCGAGCTTGGGAAGGCCTTTGAGCTGACCAAAGCAGCCGGACTGATCGAGGTGGCAGAAACGACCCTTCTGCTTCACGACAAACTTGAGAGCATCGTTATCGCAGTCAAGCGAGATGCGGACGAGCTCCTGAGTGTCTCCGGAAGTAGCACCCTTGTACCAGAGACCGCGCTTCCGGCTCTGATAGACACCAGTCTGTGTCCTGAGGGCCTCGTTCACACTCTCGGCACTGCTGTAGACCAGACCCAGAGCAGTGTCGTGCTCATCAACGACAACGGTGGGGAGAAGACCATCGGGACGGTCAGACTTCCAGACGCTCGAGAGGATGGTGGAGATGGCAAGCTTCAGCTGCTCGAGTTC
