# Supplementary figures and images for: Trichophyton rubrum LysM proteins bind to fungal cell wall chitin and to the N-linked oligosaccharides present on human skin glycoproteins
Source: PLoS One. 2019 Apr 4;14(4):e0215034. doi: 10.1371/journal.pone.0215034 (PMC6449025; doi:10.1371/journal.pone.0215034)

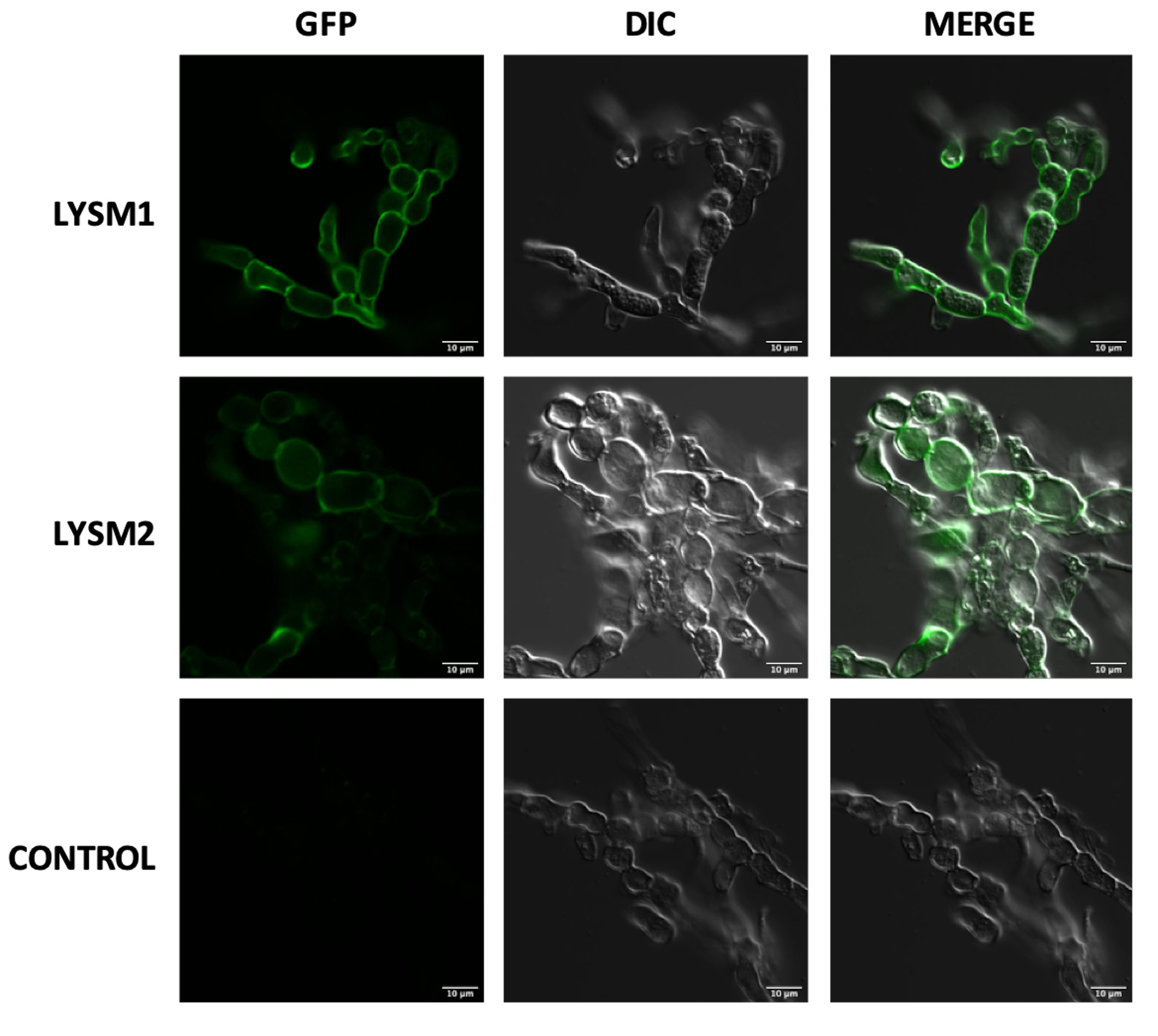

Supplement: S3 Fig — Cells from a transformant expressing LysM1::GFP (top row), from a transformant expressing LysM2::GFP (middle row), and from the non-transformed parental strain (bottom row) were examined by confocal microscopy. The left panels show the GFP images, the middle panels show the DIC images, and the right panels show the merged images. (TIFF) [file pone.0215034.s004.tiff]

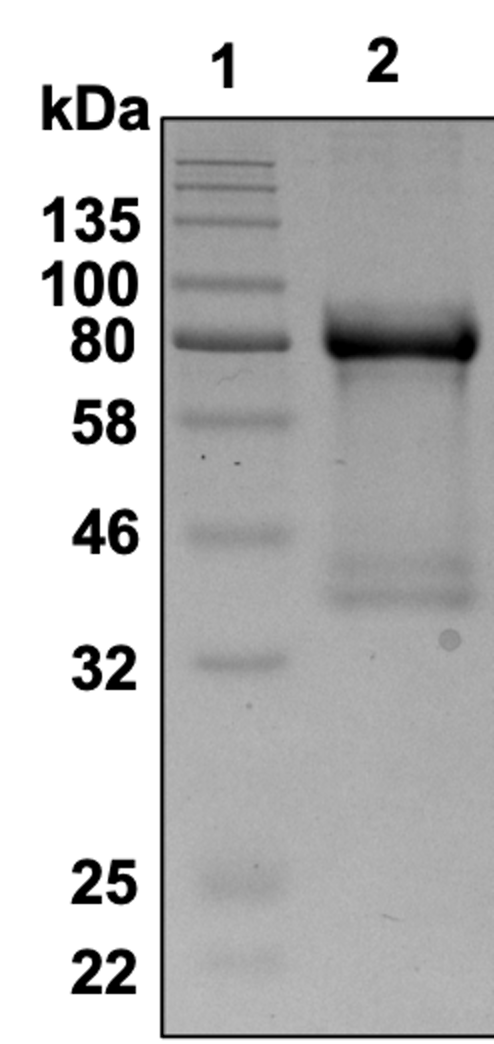

Supplement: S4 Fig — Lane 1 contains molecular weight markers and lane 2 contains the amylose resin purified chimeric MBP::LysM2 protein. (TIFF) [file pone.0215034.s005.tiff]

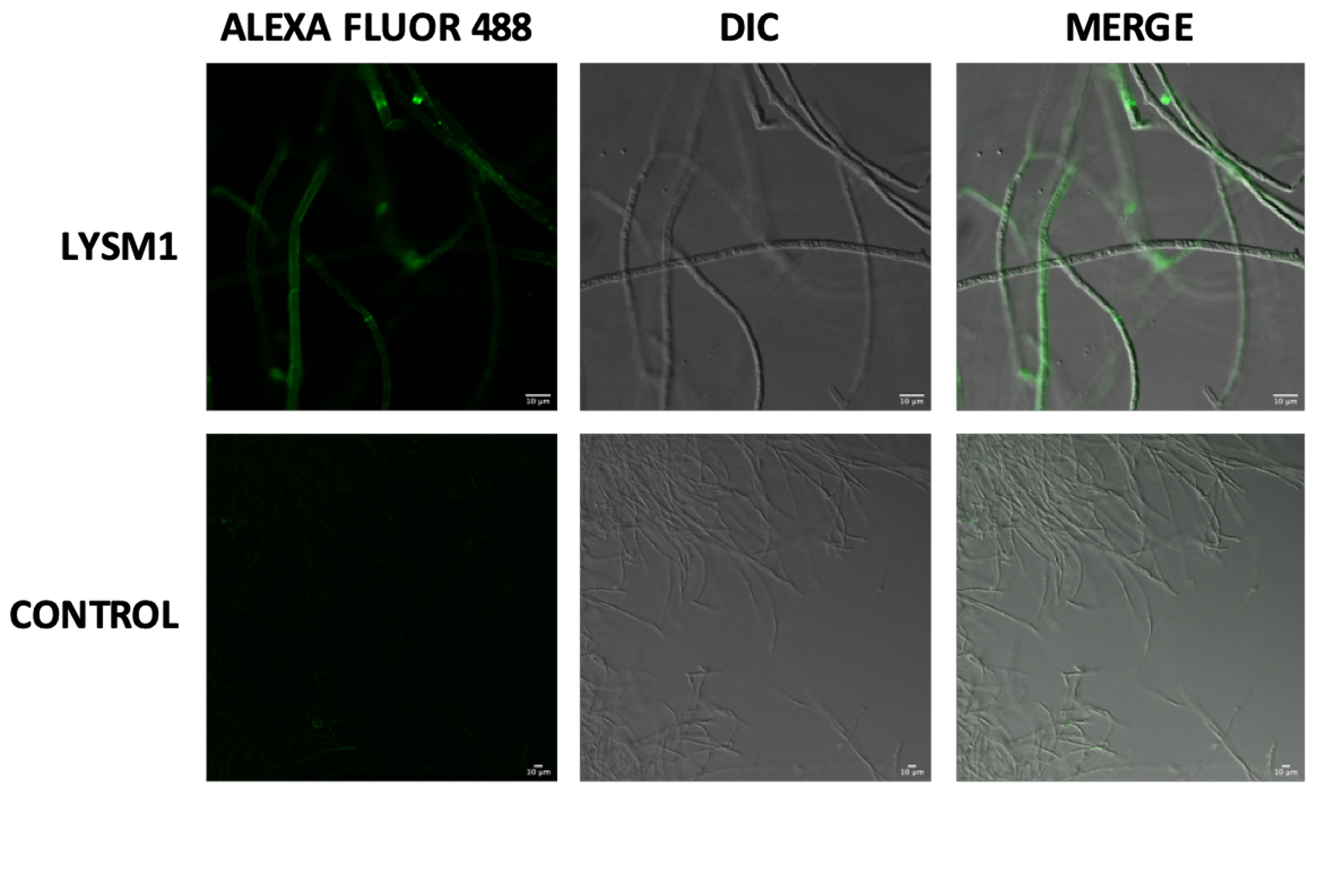

Supplement: S5 Fig — N. crassa hyphae were incubated with purified MBP::LysM1 and the binding of LysM1 to the cells was visualized with a fluorescent antibody protocol using antibody directed against MBP. The top row contains cells stained for the binding of MBP:: LysM1 and the bottom row contains cells strained for the binding of the MBP control. The left panels show the fluorescent images. The middle panels show the DIC images. The right panels contain the merged images. (TIFF) [file pone.0215034.s006.tiff]

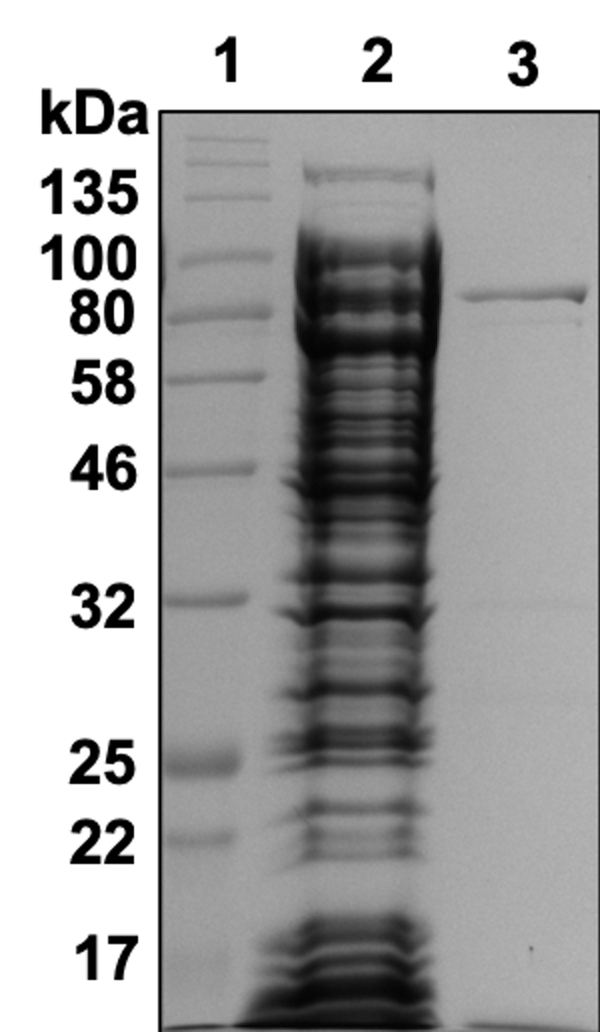

Supplement: S6 Fig — Lane 1 contains molecular weight markers. Lane 2 contains E coli lysate from a transformant expressing MBP::LysM2. Lane 3 contains the chitin binding proteins from the transformant lysate. (TIFF) [file pone.0215034.s007.tiff]
